# Discovery of entomopathogenic fungi across geographical regions in southern China on pine sawyer beetle *Monochamus alternatus* and implication for multi-pathogen vectoring potential of this beetle

**DOI:** 10.3389/fpls.2022.1061520

**Published:** 2022-12-28

**Authors:** Shengxin Wu, Jia Wu, Yun Wang, Yifei Qu, Yao He, Jingyan Wang, Jianhui Cheng, Liqin Zhang, Chihang Cheng

**Affiliations:** ^1^ School of Forestry and Biotechnology, Zhejiang A&F University, Hangzhou, Zhejiang, China; ^2^ Key Laboratory of Vector Biology and Pathogen Control of Zhejiang Province, Huzhou University, Huzhou, Zhejiang, China; ^3^ Station of Forest Pest Control, Anji Forestry Bureau, Huzhou, Zhejiang, China

**Keywords:** entomopathogenic fungi, *Monochamus alternatus*, cross-latitudinal, multi-pathogen vector, *Pinus massoniana*, biological control

## Abstract

Entomopathogen-based biocontrol is crucial for blocking the transmission of vector-borne diseases; however, few cross-latitudinal investigations of entomopathogens have been reported for vectors transmitting woody plant diseases in forest ecosystems. The pine sawyer beetle *Monochamus alternatus* is an important wood borer and a major vector transmitting pine wilt disease, facilitating invasion of the pinewood nematode *Bursaphelenchus xylophilus* (PWN) in China. Due to the limited geographical breadth of sampling regions, species diversity of fungal associates (especially entomopathogenic fungi) on *M*. *alternatus* adults and their potential ecological functions have been markedly underestimated. In this study, through traditional fungal isolation with morphological and molecular identification, 640 fungal strains (affiliated with 15 genera and 39 species) were isolated from 81 beetle cadavers covered by mycelia or those symptomatically alive across five regional populations of this pest in southern China. Multivariate analyses revealed significant differences in the fungal community composition among geographical populations of *M*. *alternatus*, presenting regionalized characteristics, whereas no significant differences were found in fungal composition between beetle genders or among body positions. Four region-representative fungi, namely, *Lecanicillium attenuatum* (Zhejiang), *Aspergillus austwickii* (Sichuan), *Scopulariopsis alboflavescens* (Fujian), and *A*. *ruber* (Guangxi), as well as the three fungal species *Beauveria bassiana*, *Penicillium citrinum*, and *Trichoderma dorotheae*, showed significantly stronger entomopathogenic activities than other fungi. Additionally, insect-parasitic entomopathogenic fungi (*A*. *austwickii*, *B*. *bassiana*, *L*. *attenuatum*, and *S*. *alboflavescens*) exhibited less to no obvious phytopathogenic activities on the host pine *Pinus massoniana*, whereas *P*. *citrinum*, *Purpureocillium lilacinum*, and certain species of *Fusarium* spp.—isolated from *M*. *alternatus* body surfaces—exhibited remarkably higher phytopathogenicity. Our results provide a broader view of the entomopathogenic fungal community on the vector beetle *M*. *alternatus*, some of which are reported for the first time on *Monochamus* spp. in China. Moreover, this beetle might be more highly-risk in pine forests than previously considered, as a potential multi-pathogen vector of both PWN and phytopathogenic fungi.

## Introduction

Vector-borne diseases occur in humans and agro-ecosystems, causing severe global health problems and economic losses. In recent decades, a variety of human and animal diseases transmitted by mosquitoes and ticks (Zellner and Huntley, 2019; Gao et al., 2020), together with notorious crop pathogen outbreaks vectored by destructive agricultural pests such as psyllids, thrips, and whiteflies (Navas-Castillo et al., 2011; He et al., 2020; Moreno et al., 2021), have increased the demand for advanced vector control technologies and strategies. Due to the environmental risks of excessive use of chemical pesticides and vector insecticidal resistance, sympatric natural enemies of vectors, including parasitoids, predatory arthropods, and entomopathogenic microbes, were explored in their distribution areas at cross-latitudinal scales (Garcia et al., 2020; Nechols, 2021), some of which have been newly discovered and developed as effective, safe, and economically acceptable alternatives to chemical control (Wang et al., 2019; Islam et al., 2021). Compared to other biocontrol agents, features, such as easy multiplication, host specificity, and high survival in varied environments, of natural and gene-engineered entomopathogenic fungi are more favorably adopted to supplement the arsenal of biological control to manage medical and agricultural insect vectors (Leger and Wang, 2010; Fang et al., 2012; Javed et al., 2019; Lovett et al., 2019).

Forest insect pests, especially wood borers, carry the assembly of phytopathogens or have tight relationships with specific associates that amplify the negative effects of their insect hosts or even dominate significant forest collapses (Linnakoski and Forbes, 2019). For example, bark beetle *Scolytus multistriatus*, a vector of the pathogen *Ophiostoma ulmi*, causes Dutch Elm Disease (Jürisoo et al., 2021), and the wood wasp *Sirex noctilio* transmits several phytopathogenic fungi (Corley et al., 2019). Moreover, a large number of forest insect pests have intimate connections with tree pathogens as potential vectors (Humble and Allen, 2006; Hulcr and Dunn, 2011). However, few cross-latitudinal investigations have been conducted on the distribution and diversity of entomopathogenic fungal species of insect vectors transmitting important woody plant diseases in natural forest ecosystems, substantially restricting the resource exploration of promising entomopathogenic fungi. At present, a strikingly limited number of entomopathogenic fungal species (e.g., *Beauveria bassiana* and *Metarhizium anisopliae*) constitutes the main microbial biocontrol agents for application in natural fields, especially in forest ecosystems (Dara et al., 2019; Rajula et al., 2020; Shehzad et al., 2021; Shehzad et al., 2022), which would be insufficient to encounter the increasing challenges from both indigenous species outbreaks and exotic species invasions.

The stem-borer of pine trees, *Monochamus alternatus*, not only bores into branches and trunks of host pine to hinder the transportation of nutrients and water, but also facilitates the invasion of plant-parasitic pinewood nematode (PWN, *Bursaphelenchus xylophilus*) as the main vector beetle in pine forest systems in China and adjacent countries (Kobayashi et al., 1984; Futai, 2013; Zhao and Sun, 2017). Investigations of natural entomopathogenic fungi for *Monochamus* spp. in Spain, Japan, and Anhui/Zhejiang Province of China showed very similar results, demonstrating that *Beauveria* species were the most abundant isolates, followed by *Metarhizium* or *Lecanicillium* (*Verticillium*) (Shimazu, 2004; Han et al., 2007; Ma et al., 2009; Álvarez-Baz et al., 2015). In contrast to the middle-high latitudinal regions covered by previous surveys, low latitudinal regions, especially southern China, are habitats where pine wilt disease (PWD, caused by PWN) initially emerged and lasts for long time (Tang et al., 2021). Considering that higher fungal diversity occurs in regions with high temperature and humidity and that several kinds of natural enemies evolve relationships with target insects over a longer period, the cross-latitudinal regions in southern China are considered an ideal and rich resource reservoir for exploring novel entomopathogenic fungi with previously unknown functions in the biocontrol of PWD transmission. However, few studies have been conducted in this area.

To explore underlying natural resource of entomopathogenic fungi on *M*. *alternatus* from low latitudinal regions of southern China, in the present study, we firstly investigated the diversity and community composition of fungal species associated with naturally infected *M*. *alternatus* across five geographical populations in southern China and assessed their relationships with the latitudinal regions. Then, we evaluated entomopathogenic activities and infection phenotypes of representative fungal isolates, followed by measurements of compatibility of these fungi with the host pine *P*. *massoniana*. Results showed that variation of fungal community composition significantly couples with the geographical origin of naturally infected *M*. *alternatus* and the main fungal species from each population are region-specific. Enzymatic and *in vivo* interactive bioassays combined with morphological and molecular identifications revealed strongly entomopathogenic fungi functioning in parasitic or non-parasitic mode, which are well compatible with host pine. These findings provide new insights into the distribution of *M*. *alternatus* entomopathogenic fungi across geographical regions as well as their promising application in the field to break down transmission of the pine wilt disease and potentially vectoring phytopathogenic fungi by the beetle.

## Materials and methods

### Beetle sampling, fungal isolation, and fungal identification

The pine sawyer beetle *M*. *alternatus* was collected using commercial traps (FEILUOMENG Co., China) baited with attractants in naturally infested pine forests from five geographical regions of southern China (**Figure S1**). The specimens were labeled and transported to the laboratory in individual sterilized tubes for each beetle with fresh twigs. During rearing of the collected beetles (approximately 500 beetles in total), 81 beetle cadavers or symptomatically living ones with fungal infections were observed and stored at 4°C for use in this study.

Fungal isolation was performed under aseptic conditions following previously described procedures (Parker et al., 2003). The beetle body was divided into seven positions using sterilized scissors: antennae, head, thorax, abdomen, eggs (for females), wings, and legs. Tissues were inoculated onto potato dextrose agar (PDA) plates containing 0.05 g/L streptomycin, penicillin G and tetracycline and then cultured in an incubator at 25°C until the tissues were covered and surrounded by mycelia. Fungal purification was repeated twice on PDA with antibiotics, and pure isolates were cultured on conventional PDA and deposited in the Key Laboratory of Vector Biology and Pathogen Control of Zhejiang Province at Huzhou University.

The fungal isolate was cultured in 50 ml sterile potato dextrose broth (PDB) at 25°C with shaking at 180 rpm in a 250ml flask for 5–7 days. Mycelia harvested from the PDB were separated by filtration and homogenized in liquid nitrogen using a pre-cooled mortar and pestle. Genomic DNA was extracted using the Rapid Fungi Genomic DNA Isolation Kit (Sangon Biotech Co., China), and the rDNA-ITS region was amplified using primer pairs ITS1 and ITS4 (White et al., 1990) *via* the following procedure: initial denaturing at 95°C for 4 min, followed by 35 cycles of denaturing at 94°C for 60 s, annealing at 58°C for 60 s, elongation at 72°C for 2 min, and a final elongation at 72°C for 10 min. Amplified PCR products were sequenced and compared with the ITS sequence database in GenBank using BLAST software on the National Center for Biotechnology Information (NCBI) website. The gene sequences were deposited in NCBI under GenBank accession numbers OP321299–OP321543.

### Evaluation of entomopathogenic activities for representative fungi isolated from geographical populations of *M*. *alternatus*

Based on the above results, eleven representative fungal species, namely, *Aspergillus austwickii*, *A*. *ruber*, *Beauveria bassiana*, *Clonostachys rosea*, *Lecanicillium attenuatum*, *L*. *aphanocladii*, *Penicillium citrinum*, *Pestalotiopsis disseminata*, *Purpureocillium lilacinum*, *Scopulariopsis alboflavescens*, and *Trichoderma dorotheae*, were used to test their entomopathogenic activities. Considering that it is difficult to collect an adequate quantity of adult *M*. *alternatus* with long-lasting alive status from the field to match this bioassay, the population of the model insect beetle *Tribolium castaneum*, successfully reared in the laboratory with a common genetic background, was used to determine the entomopathogenicity of the eleven fungal isolates. Their infection phenotypes on *M*. *alternatus* were also further confirmed in the subsequent experiments. Conidia obtained from 14-day-old fungi were suspended in a sterile aqueous solution of 0.01% Tween-80 (Orduño-Cruz et al., 2015). This suspension was shaken with glass beads and filtered through two layers of gauze to remove the mycelia. The conidia were counted with a hemocytometer and adjusted to 1×10^8^ conidial/ml with sterile 0.01% Tween-80.

Adult *T*. *castaneum* was reared on wheat bran at 25°C in a climate-controlled incubator. Beetles were surface-sterilized with bleach, ethanol, and distilled water [10:10:80 (vol:vol)] and starved for 24 h before use. Aliquots of wheat bran were UV-sterilized, placed in sterile Petri dishes, and then treated with conidial suspension. *T*. *castaneum* beetles were also immersed in the conidial suspension for 10 s and transferred to a Petri dish (n = 20 in each Petri dish). The wheat bran was replaced with a new conidial treatment every 4 days. Beetles and wheat bran in the control group were treated with sterile 0.01% Tween-80 solution. Each fungal and control group was replicated three times. In a pilot assay, beetles were checked for mortality 9 days after fungal treatments and in the subsequent survival monitoring assay, beetles were recorded daily for 15 days. Dead beetles from these assays were transferred to sterile moist centrifugal tubes and inspected for fungal growth on the cadavers. The aerial hyphae of each fungal species on cadavers were confirmed using Koch’s postulate.

Fungal entomopathogenic activity correlated well with the activities of three enzymes including protease, chitinase, and lipase, in *M*. *alternatus* and many other insect pests (Cortez-Madrigal et al., 2014; Dhawan and Joshi, 2017; Moharram et al., 2021). The enzyme activities of the fungal species in this study were measured following previously reported procedures (Sharma et al., 2016; Li et al., 2018; Alabdalall et al., 2020) with minor modifications. To test protease activity, conidia (1×10^8^ conidia/ml) of each isolated fungus were cultured in 50 ml of protease-inducing medium with shaking at 180 rpm at 25°C for 10 days. One milliliter of fermentation supernatant was obtained from each culture solution every 2 days and incubated with 1 ml of 1% casein solution at 37°C for 30 min. The quantities of the reaction products were determined by measuring the optical density at 600 nm (OD600) using the Folin-phenol reagent method. The negative control of each sample was prepared by adding trichloroacetic acid to the reaction solution before incubation to inhibit putative enzymatic activities. Five concentrations of tyrosine were used to create a standard curve, and one protease unit was defined as the amount of fermentation supernatant required to produce 1 µg of tyrosine from casein per minute. To test chitinase activity, conidia (1×10^8^ conidia/ml) of fungi were cultured in liquid chitin medium for 10 days and at every 2 d-interval, 1 ml of fermentation supernatant was incubated with 1 ml of 1% colloidal chitin at 50°C for 1 h. The negative control for each sample was prepared by heating the reaction solution in a boiling water bath before incubation. Reaction product quantities were determined by measuring the OD540 using the 3,5-dinitrosalicylic acid (DNS) method. Five concentrations of N-acetyl-D-glucosamine were used to create a standard curve, and one chitinase unit was defined as the amount of fermentation supernatant required to produce 1 µg of N-acetyl-D-glucosamine per minute. To test lipase activity, conidia (1×10^8^ conidia/ml) of fungi were cultured in Sabouraud’s dextrose agar (SDA) medium for 10 days and 0.2 ml of fermentation supernatant was pipetted every 2 days and incubated with 0.2 ml matrix solution containing 4-nitrophenyl palmitate (Gopinath et al., 2005) at 37°C for 30 min. The negative control for each sample was prepared by adding trichloroacetic acid to the reaction solution before incubation. The quantity of the reaction product *p*-nitrophenol was calculated by comparing the OD410 value with the linear standard curve, and one lipase unit was defined as the amount of fermentation supernatant required to produce 1 µg of *p*-nitrophenol per minute. Three biological replicates were used for enzymatic bioassays.

### Morphological observation and phylogenetic analyses of fungal species with entomopathogenic activities

The fungal species with remarkable entomopathogenic activities were cultured on PDA plates for 7–11 days at 25°C. Circular agar blocks (5 mm in diameter) from colonies were transferred to new PDA plates to observe colony morphology. For asexual morphological descriptions, conidia, conidiophores and nutritional hyphae were sampled from colonies on glass slides, and their traits were observed and measured using a Leica DM2000 microscope (Leica Co., Germany).

Fungal infection phenotypes in live *M*. *alternatus* adults (sampled from Zhejiang Province) were confirmed following the procedure described above. Beetles were surface-sterilized, dipped in conidia suspension, transferred to 50-ml sterile tubes (one beetle per tube), and provided with fresh pine twigs (also immersed in conidia suspension for 10 s). Beetle activity was assessed based on the quantity of the frass produced. When feeding ceased, the beetle was transferred to a new 50-ml tube (with sterile moist cotton) for fungal growth for 7 days. Each fungal species was applied to five living beetles. The infected beetles were observed as described above and fulfilled by Koch’s postulates.

For scanning electron microscopy (SEM) observation, colony plugs and infected beetles were fixed in pre-chilled 2.5% glutaraldehyde at 4°C for 2 days. Samples were washed thrice in 0.1% phosphate buffer (pH 7.2–7.4) for 5 min and dehydrated in 30%, 50%, 70%, 80%, 90%, 95%, 100% ethanol for 10 min. The samples were dried in a vacuum freeze dryer (Yamato Scientific Co., Japan), coated with platinum using a sputter coater, and observed using an S-3400N scanning electron microscopy (Hitachi, Japan).

The primer pairs NS1/NS4 (White et al., 1990), LR7/LROR (Vilgalys and Hester, 1990; Rehner and Samuels, 1994), EF-983F/EF-2218R (Aphidech and Kusavadee, 2013), RPB2-5’F/RPB2-5’R (Wang et al., 2015), and TUB1/TUB22 (Qi et al., 2021), were used to amplify a region spanning of the nuclear ribosomal *SSU* gene, a segment of the large subunit rRNA gene (*LSU*), part of the elongation factor 1-alpha (*EF-1α*) gene, the second largest subunit sequences of RNA polymerase ІІ (*rpb2*), and part of the *β-tubulin* gene, respectively. The PCR products were examined by 1.5% agarose gel electrophoresis and then subjected to sequencing with the GenBank accession numbers listed in **Table S1**. Sequences of ITS and the five genes were then aligned using Clustal X2.0 and MEGA 6 (Larkin et al., 2007; Tamura et al., 2013). Ambiguously aligned sites were excluded, and gaps were treated as missing data during sequence alignment. The aligned sequences of the six genes were concatenated to construct Maximum Likelihood (ML) phylogenetic trees using MEGA 6.

### Detection of phytopathogenic activities to *Pinus massoniana* by the entomopathogenic fungi

Two- to three-year-old *P*. *massoniana* seedlings were used to determine the phytopathogenic activities of the entomopathogenic fungi. Fungal inoculation was conducted by making a wound on the main stem of each *P*. *massoniana* seedling using a sterile cork borer with a 5-mm diameter at 15 cm above the soil line (one seedling with one inoculation point). A plug with a 5-mm diameter was taken from the margin of one actively growing fungal species cultured on PDA and transferred to the cambium layer of seedling inoculation point. The inoculation points were wrapped in a laboratory film (Parafilm M, USA) to prevent contamination and desiccation. Mock inoculation, mimicked using a plug of PDA alone (without fungi), was applied to the seedlings in the same manner as a control. The *Fusarium* species, which are broad-spectral plant pathogens, together with other non-entomopathogenic fungi were also included in the experiment. Each fungal isolate and the control were replicated thrice. After 3 weeks, lesion length was measured both downward and upward from the inoculation point. The fungi were re-isolated from the infected areas to complete Koch’s postulates.

To measure pectinase and cellulase activities, conidia (1×10^8^ conidia/ml) of each fungus were cultured in 50 ml of pectinase- or cellulase-inducing medium (0.2% KNO_3_, 0.05% KCl, 0.001% FeSO_4_, 0.1% K_2_HPO_4_, 0.05% MgSO_4_·7H_2_O, 1% pectin, or 1% carboxymethyl cellulose sodium; pH 5.0) for 10 days. For every 2 days, 1 ml of fermentation supernatant was pipetted and incubated with 1 ml of 0.1% pectin solution or 0.5% sodium carboxymethyl cellulose solution at 50°C for 1 h. The negative control for each sample was prepared by heating the reaction solution in a boiling water bath for 5 min before incubation. After incubation, the reaction solutions were bathed in boiling water, incubated with 2 ml of DNS, boiled again at 100°C for 5 min, and finally refrigerated on ice. Reaction product quantities were determined by measuring the OD540 using the 3,5-dinitrosalicylic acid (DNS) method (Pereira et al., 2002). Five concentrations of D-galacturonic acid and D-glucose were used to generate the standard curves. One pectinase unit and one cellulase unit were defined as the amounts of fermentation supernatant to produce 1 µg of D-galacturonic acid and 1 µg of D-glucose per minute, respectively. The enzymatic bioassays were performed in triplicate.

### Statistical analysis

Variations in α-diversity indices of fungal communities among geographical regions, mortality of beetles caused by fungi at 9 d, enzymatic activity levels, and phytopathogenicity among fungi were assessed using one-way ANOVA followed by the Bonferroni approach for pair-wise comparisons. Where normality and/or equal variance were not assumed, nonparametric Kruskal-Wallis one-way ANOVAs were performed, followed by pair-wise comparisons using the Mann-Whitney *U* test. Absolute abundance was estimated as the number of isolates per fungal taxa, whereas the ratio of isolates from each fungal taxa to total fungal isolates was considered the relative abundance of certain taxa. Venn diagrams and upset plots were used to display the intersection of fungal species in beetle geographical populations (or body positions or genders) using R software with the package *ggplots2*. Principal component analysis (PCA) was used to investigate the variation patterns of the fungal community structures among geographical locations. Sörensen’s similarity index (Cs) was calculated using the equation: ( $\text{Cs}=\frac{2c}{a+b}$ ) (Sörensen, 1948), where a and b are the numbers of species unique to each geographical location and c is the number of shared species between the two locations. Principal coordinate analysis (PCoA) was applied to visualize the relationship between the variation in fungal community composition and beetle geographical populations (or body positions or genders), followed by significance tests using one-way PERMANOVA. The survival of beetles was calculated using Kaplan-Meier survival analysis. Comparisons between survival curves were further tested using the Log Rank (Mantel-Cox) method. GraphPad Prism 6 and PAST software were used for statistical analyses.

## Results

### Community compositions of fungal associates vary significantly among geographical populations of *M*. *alternatus* with natural fungal infections

A total of 640 fungal strains were isolated from 81 beetle cadavers or those symptomatically alive in five geographical regions, belonging to 15 fungal genera and 39 species (**Table 1**). Genus *Aspergillus* was highest in relative abundance (35.47%), followed by *Penicillium* (25.31%), *Scopulariopsis* (9.69%), *Lecanicillium* (8.75%), *Fusarium* (7.66%), and *Trichoderma* (6.42%). These genera accounted for 93.30% of the total identified strains. The most dominant species was *A*. *ruber* (21.72%), followed by *P*. *citrinum* (9.84%), *A*. *sydowii* (9.84%), *S*. *alboflavescens* (9.69%), *L*. *attenuatum* (8.44%), and *F*. *annulatum* (5.16%). These collectively represented 64.69% of the total identified strains.

**Table 1:** GenBank accession numbers of fungal isolates from *M*. *alternatus* in this study and similarity scores to closest (type) strains in NCBI according to the rDNA-ITS region.

Species affiliation	GenBank accession no.	Closest (type) strains	Similarity	No. of isolates	RA(Species)^a^	RA(Genus)^b^
*Aspergillus austwickii*	OP321299-OP321307	*Aspergillus austwickii* (NR_171607)	100 (573/573)	25	3.91%	35.47%
*Aspergillus ruber*	OP321308-OP321337	*Aspergillus ruber* (NR_131286)	100 (523/523)	139	21.72%	
*Aspergillus sydowii*	OP321338-OP321348	*Aspergillus sydowii* (NR_131259)	100 (510/510)	63	9.84%	
	OP321349-OP321355		99.80 (510/511)			
	OP321356-OP321358		99.41 (507/510)			
*Arthrinium rasikravindrae*	OP321359	*Arthrinium rasikravindrae* (NR_119932)	99.65 (577/579)	1	0.16%	0.16%
*Beauveria bassiana*	OP321360-OP321361	*Beauveria bassiana*(NR_111594)	99.24 (523/527)	2	0.31%	0.31%
*Clonostachys aranearum*	OP321362-OP321369	*Clonostachys aranearum* (NR_164542)	99.81 (538/539)	10	1.56%	2.66%
	OP321370		99.63 (541/543)			
*Clonostachys eriocamporesiana*	OP321371	*Clonostachys eriocamporesiana*(NR_168235)	100 (480/480)	4	0.63%	
*Clonostachys rosea*	OP321372- OP321374	*Clonostachys rosea* (EU326187)	100 (543/543)	3	0.47%	
	OP321375	*Clonostachys rosea* (MK713423)	99.82 (550/551)			
*Cladosporium delicatulum*	OP321376- OP321377	*Cladosporium delicatulum* (MT548673)	100 (524/524)	1	0.16%	0.16%
*Fusarium annulatum*	OP321378- OP321388	*Fusarium annulatum* (NR_138275)	100 (534/535)	33	5.16%	7.66%
*Fusarium circinatum*	OP321389	*Fusarium circinatum* (NR_120263)	98.85 (517/523)	2	0.31%	
*Fusarium foetens*	OP321390	*Fusarium foetens* (NR_159865)	99.41 (508/511)	12	1.88%	
	OP321391- OP321392		99.22 (507/511)			
*Fusarium polyphialidicum*	OP321393- OP321394	*Fusarium polyphialidicum* (MT422090)	100 (527/527)	2	0.31%	
	OP321395		99.61 (517/519)			
*Lecanicillium attenuatum*	OP321396- OP321408	*Lecanicillium attenuatum* (MH231313)	100 (570/570)	54	8.44%	8.75%
*Lecanicillium aphanocladii*	OP321409	*Lecanicillium aphanocladii* (MN511328)	100 (568/568)	2	0.31%	
*Nigrospora camelliae-sinensis*	OP321410	*Nigrospora camelliae-sinensis* (NR_153473)	97.86 (502/513)	1	0.16%	0.32%
*Nigrospora musae*	OP321411	*Nigrospora musae* (NR_153478)	(521/522)	1	0.16%	
*Penicillium cairnsense*	OP321412- OP321438	*Penicillium cairnsense* (NR_121508)	99.82 (567/568)	32	5.00%	25.31%
*Penicillium citrinum*	OP321439- OP321452	*Penicillium citrinum* (NR_121224)	100 (525/525)	63	9.84%	
*Penicillium crustosum*	OP321453	*Penicillium crustosum* (NR_077153)	100 (563/563)	1	0.16%	
*Penicillium chrysogenum*	OP321454- OP321455	*Penicillium chrysogenum* (NR_077145)	100 (569/569)	7	1.09%	
*Penicillium meleagrinum* var. *viridiflavum*	OP321456- OP321462	*Penicillium meleagrinum* var. *viridiflavum* (NR_153214)	100 (516/516)	22	3.44%	
	OP321463- OP321465		99.81 (516/517)			
	OP321466		99.61 (516/518)			
	OP321467- OP321469		100 (545/545)			
*Penicillium ochrochloron*	OP321470- OP321471	*Penicillium ochrochloron* (NR_111509)	99.82 (544/545)	10	1.56%	
*Penicillium quebecense*	OP321472- OP321479	*Penicillium quebecense* (NR_121507)	99.82 (562/563)	27	4.22%	
	OP321480		99.64 (560/562)			
*Pestalotiopsis disseminata*	OP321481	*Pestalotiopsis disseminata* (MK862235.1)	100 (577/577)	1	0.16%	1.26%
*Pestalotiopsis microspora*	OP321482	*Pestalotiopsis microspora* (MK801280)	100 (533/533)	6	0.94%	
*Pestalotiopsis grevilleae*	OP321483	*Pestalotiopsis grevilleae* (NR_147548)	99.31 (574/578)	1	0.16%	
*Purpureocillium lilacinum*	OP321484- OP321485	*Purpureocillium lilacinum* (NR_165946)	100 (561/561)	5	0.78%	0.78%
	OP321486- OP321487		99.64 (560/562)			
*Scopulariopsis alboflavescens*	OP321488- OP321499	*Scopulariopsis alboflavescens* (NR_156620)	98.17 (590/601)	62	9.69%	9.69%
	OP321500- OP321502		97.84 (589/602)			
	OP321503- OP321504		97.67 (588/602)			
*Syncephalastrum monosporum* var. *pluriproliferum*	OP321505	*Syncephalastrum monosporum* var. *pluriproliferum* (NR_160185)	98.78 (162/164)	1	0.16%	0.16%
*Trichoderma atroviride*	OP321506- OP321507	*Trichoderma atroviride* (NR_077207)	100 (572/572)	3	0.47%	6.42%
*Trichoderma appalachiense*	OP321508	*Trichoderma appalachiense* (NR_134340)	99.49 (583/586)	1	0.16%	
*Trichoderma dorotheae*	OP321509- OP321510	*Trichoderma dorotheae* (NR_166014)	100 (576/576)	23	3.59%	
	OP321511- OP321523		99.83 (576/577)			
	OP321524		99.48 (574/577)			
*Trichoderma hispanicum*	OP321525	*Trichoderma hispanicum* (NR_138451**)**	100 (576/576)	1	0.16%	
*Trichoderma lixii*	OP321526- OP321529	*Trichoderma lixii* (NR_131264)	99.83 (591/592)	5	0.78%	
	OP321530- OP321533		99.66 (594/596)			
	OP321534		99.50 (593/59))			
	OP321535		99.49 (589/592)			
*Trichoderma neokoningii*	OP321536	*Trichoderma neokoningii* (NR_138446)	99.48 (574/577)	4	0.63%	
*Trichoderma texanum*	OP321537	*Trichoderma texanum* (NR_137308)	100 (568/568)	4	0.63%	
*Talaromyces coalescens*	OP321538	*Talaromyces coalescens* (NR_120008)	98.65 (586/594)	1	0.16%	0.94%
*Talaromyces wortmannii*	OP321539- OP321541	*Talaromyces wortmannii* (NR_172039)	100 (584/584)	5	0.78%	
	OP321543- OP321543		99.65 (572/574)			

a Relative abundance (RA; %) was calculated as the ratio of the number of isolates of each species to that of total fungal isolates;

b Relative abundance (RA; %) was calculated as the ratio of the number of isolates of each Genus to that of total fungal isolates.

Significant differences in fungal community composition were found among geographical regions/provinces in which *M*. *alternatus* adults were sampled. In Zhejiang, a total of 197 fungal strains were isolated from the beetles, belonging to 11 genera and 22 species, in which fungal species *L*. *attenuatum* and *P*. *citrinum* were dominant, with relative abundances of 25.38% and 14.72%, respectively (**Table S2**; **Figure S2**). In Sichuan, a total of 36 fungal strains were isolated, belonging to 6 genera and 12 species, in which fungal species *A*. *austwickii* and *T*. *dorotheae* were dominant, with relative abundances of 41.67% and 19.44%, respectively (**Table S2**; **Figure S2**). In Fujian, 172 fungal strains belonging to 12 species and 6 genera were isolated, in which fungal species *S*. *alboflavescens* and *A*. *sydowii* were higher in relative abundances than others, at 31.40% and 29.07%, respectively (**Table S2**; **Figure S2**). In Guangdong, 74 fungal strains belonging to 14 species and 9 genera were isolated, and *P*. *citrinum* with a relative abundance of 22.97%, was higher than other species (**Table S2**; **Figure S2**). In Guangxi, 161 fungal strains belonging to 6 species and 5 genera were isolated, of which *A*. *ruber* with a relative abundance of 85.09% was the dominant species (**Table S2**; **Figure S2**). The fungal species, namely, *L*. *attenuatum*, *A*. *austwickii*, *S*. *alboflavescens*, and *A*. *ruber*, appeared to be region-representative in Zhejiang, Sichuan, Fujian, and Guangxi, respectively, since their relative abundances were highest in one population while strikingly low in the others. Since *P*. *citrinum* was also abundant in Zhejiang, no region-representative species were found in Guangdong.

**Figure 1:**
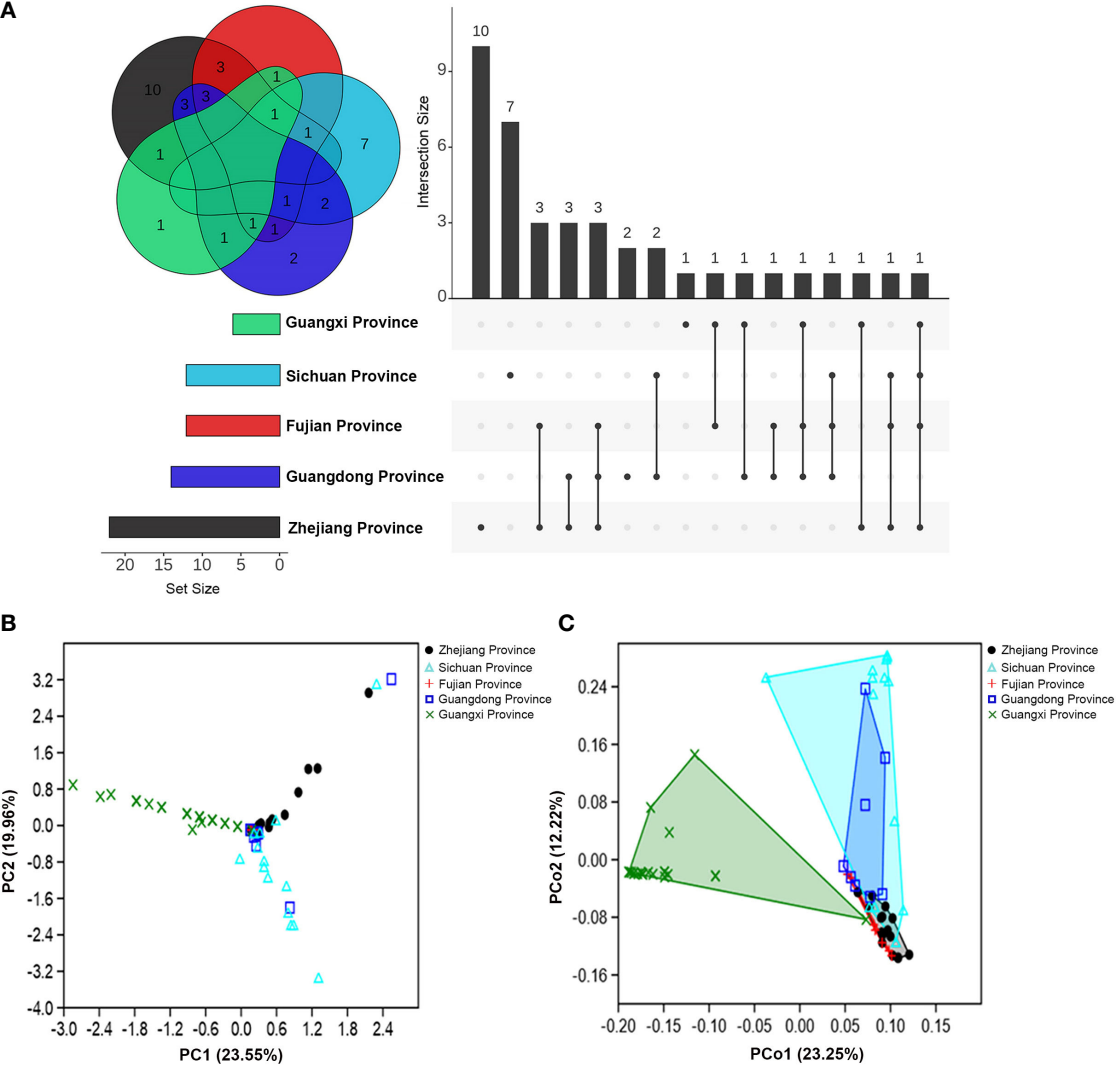
Community composition variations of fungal associates of *M*. *alternatus* populations from five geographical regions. **(A)** Venn diagram and upset plot. **(B)** PCA of Bray-Curtis distance differentiating patterns of samples from different populations according to fungal community composition. **(C)** PCoA of Bray-Curtis distance showing variation in fungal community composition among geographical populations.

Additionally, except for the fungi isolated from *M*. *alternatus* in Fujian population, fungal communities from the rest of geographical populations contained distinct species (exclusively isolated species) in their respective populations (**Figure 1A**). Furthermore, the number of shared fungal species in the Fujian and Guangdong population was higher than that in the other populations (**Figure 1A**).

The α-diversity index comparisons of fungal communities among populations and multivariate analyses further supported the geographical variation in community composition. An obvious decrease in fungal species diversity was found in high- to low-latitudinal populations (**Table S3**). Species richness and diversity indices were significantly higher in the population of Zhejiang than those of Guangxi, whereas dominance and evenness indices were higher in the Guangxi (**Table S3**). Principal component analysis (PCA) showed that fungal community compositions of *M*. *alternatus* in Zhejiang, Fujian, and Guangxi populations diverged remarkably from each other (**Figure 1B**), and the pair-wise similarity coefficient calculation further indicated that fungal communities from the three regions were dissimilar to each other, while the fungal composition of the Fujian population was more similar to the Guangdong than other populations (**Table S4**). Principal coordinate analysis (PCoA) elucidated the distances of fungal community composition between geographical population groups and inter-sample variations within groups (**Figure 1C**; one-way PERMANOVA; *F* = 9.180, *P* = 0.0001). Pair-wise comparisons also demonstrated strikingly significant mutual dissimilarities in fungal composition between different *M*. *alternatus* geographical populations, except for those isolated from Fujian and Sichuan populations, which did not show notable differences with that from Guangdong population (**Table S5**).

Fungal communities from the five geographical populations were integrated and re-categorized according to body position (**Table S6**; **Figures S3A, B**) and gender (**Table S7**; **Figures S4A, B**). However, no significant differences were found in the fungal community composition among *M*. *alternatus* body positions (one-way PERMANOVA; *F* = 1.135, *P* = 0.217) and among genders (one-way PERMANOVA; *F* = 1.167, *P* = 0.277), as visualized by PCoA analyses (**Figures S3C, S4C**).

### Main fungal species isolated from the five geographical populations of infected *M*. *alternatus* adults present strong entomopathogenic activity

**Figure 2:**
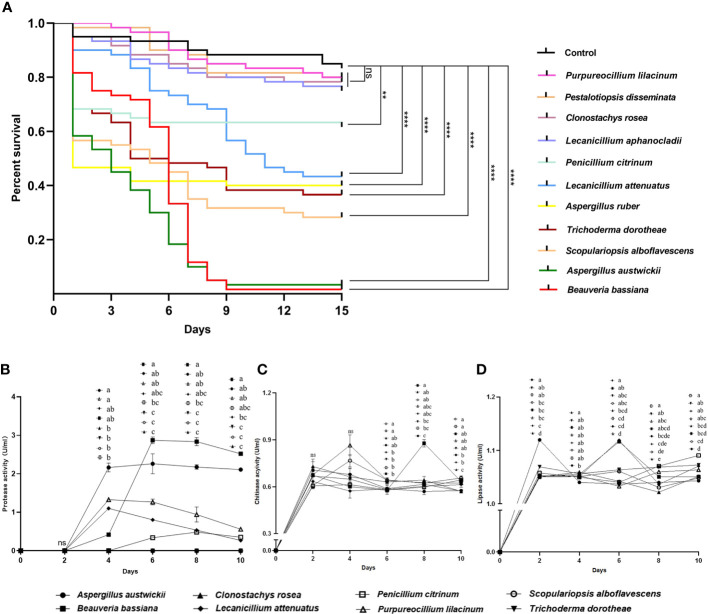
Entomopathogenic activities of representative fungal species isolated from *M*. *alternatus* populations with naturally fungal infection. **(A)** Kaplan-Meier survival curves of *T*. *castaneum* beetles inoculated with conidia suspension (1×10^8^ conidia/ml). Log-rank tests were performed and the levels of differences were denoted: ns, not significant, *P* > 0.05; **P< 0.01; ****P< 0.0001. **(B)** Protease activities of fermentation supernatants from fungal species. **(C)** Chitinase activities of fermentation supernatants from fungal species. **(D)** Lipase activities of fermentation supernatants from fungal species. In B-D, different letters mean significant differences among fungi at each time point (*P<* 0.05) and ns means not significant. Data were represented as Mean ± SD.

Fungal isolate *Beauveria bassiana*, a well-known entomopathogenic fungus, as well as four more fungal isolates with activities reported previously in insects including *Clonostachys rosea* (Mohammed et al., 2021), *L*. *aphanocladii* (Nedveckytė et al., 2021), *Pestalotiopsis disseminata* (Lv et al., 2011) and *Purpureocillium lilacinum* (Panyasiri et al., 2022), together with *A*. *austwickii*, *A*. *ruber*, *L*. *attenuatum*, *P*. *citrinum*, *S*. *alboflavescens* and *T*. *dorotheae*—which were the main species associated with their corresponding *M*. *alternatus* populations—were used to evaluate their entomopathogenic activities. In a pilot assay, after 9 days of infection, four fungal species, namely, *A*. *austwickii*, *B*. *bassiana*, *L*. *attenuatum*, and *S*. *alboflavescens* showed significantly higher mortality on the model insect beetle *T*. *castaneum* than the control (**Figure S5**; Kruskal-Wallis test; χ_10_^2^ = 25.86, *P* = 0.0039). In the subsequent 15-day-time course assay, there was a significant difference in the survival curves among treatments (**Figure 2A**; Kaplan-Meier test; χ_11_^2^ = 288.9, *P* = 0.0001). No significant differences were found between the survival of *T*. *castaneum* adults inoculated with Tween 80 (control group) and those inoculated with conidial suspension of *P*. *lilacinum*, *P*. *disseminata*, *C*. *rosea*, and *L*. *aphanocladii* (**Figure 2A**; Log-rank tests; Pl, χ_1_^2^ = 0.22, *P* = 0.6398; Pd, χ_1_^2^ = 0.44, *P* = 0.5080; Cr, χ_1_^2^ = 0.57, *P* = 0.4511; Lap, χ_1_^2^ = 1.35, *P* = 0.2456), indicating that the four fungal species did not influence beetle fitness. However, the survival of *T*. *castaneum* adults inoculated with *B*. *bassiana*, *A*. *austwickii*, *S*. *alboflavescens*, *T*. *dorotheae*, *A*. *ruber*, and *L*. *attenuatum* was significantly lower than that of the beetles in the control group (**Figure 2A**; Log-rank tests; Bb, χ_1_^2^ = 96.23, *P*< 0.0001; Aa, χ_1_^2^ = 101.6, *P*< 0.0001; Sa, χ_1_^2^ = 41.75, *P*< 0.0001; Td, χ_1_^2^ = 29.89, *P*< 0.0001; Ar, χ_1_^2^ = 26.11, *P*< 0.0001; Lat, χ_1_^2^ = 20.78, *P*< 0.0001), demonstrating the potent insecticidal activities of the six fungal species. Another fungus, *P*. *citrinum*, showed a relatively moderate efficacy in killing *T*. *castaneum* adults (**Figure 2A**; Log-rank test; χ_1_^2^ = 7.70, *P* = 0.0055).

The entomopathogenic activities of these fungal species were determined using enzymatic activity assays. *A*. *austwickii* and *B*. *bassiana* had consistently higher protease activities with incubation time than the other fungal species (**Figure 2B**). *B*. *bassiana* performed better in chitinase activity than *A*. *austwickii*, reaching a peak level higher than that of other fungi on day 8 (**Figure 2C**). Except for *B*. *bassiana*, the other fungi showed decreased chitinase activity from day 4 until the end, and *S*. *alboflavescens* exhibited a relatively higher chitinase activity during this time (**Figure 2C**). As for lipase activity, *A*. *austwickii* reached a peak level higher than other fungi on day 2 and then exhibited a decrease in activity to a stable level (**Figure 2D**). Both *B*. *bassiana* and *L*. *attenuatum* showed the highest lipase activity levels on day 6, compared to other fungi (**Figure 2D**). *P*. *citrinum* did not exhibit an increase in lipase activity until day 8–10 of the incubation (**Figure 2D**).

### Infection phenotypes are induced by the entomopathogenic fungal species on *M*. *alternatus* with their morphological characteristics comparable to those on *T*. *castaneum* and on PDA medium

**Figure 3:**
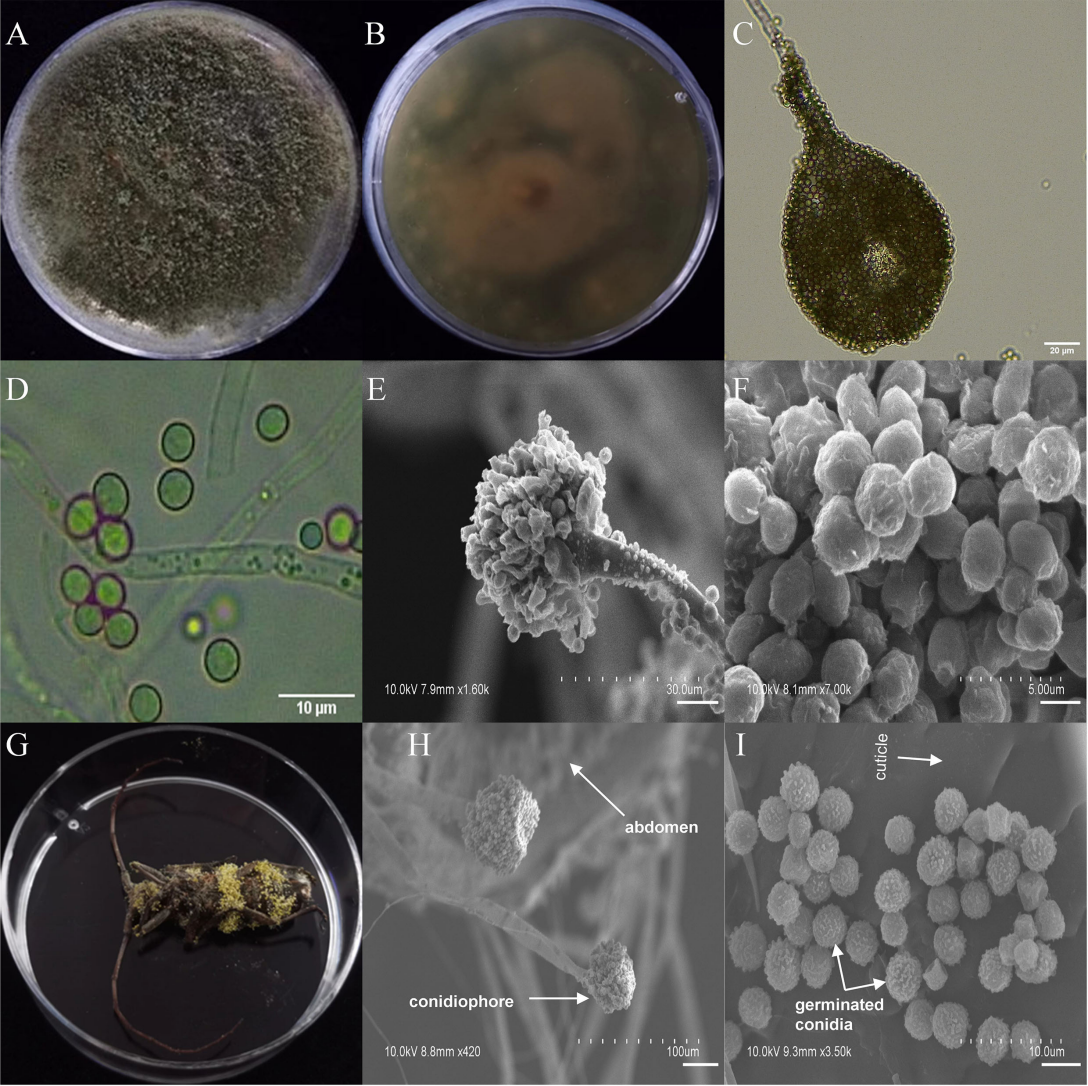
Morphology of *A*. *austwickii* and *M*. *alternatus* cadaver infected by *A*. *austwickii* under optical microscope and SEM. **(A)** Colonial morphology cultured on PDA. **(B)** Reverse of colony on PDA. **(C)** Hyphae and conidiophores (OM). **(D)** Conidia (OM). **(E)** Conidiophores (SEM). **(F)** Conidia (SEM). **(G)** *M*. *alternatus* cadaver surrounded by mycelia. **(H)** Conidiophores grown from *M*. *alternatus* cuticle (SEM). **(I)** Conidia on *M*. *alternatus* cuticle surface (SEM).

**Figure 4:**
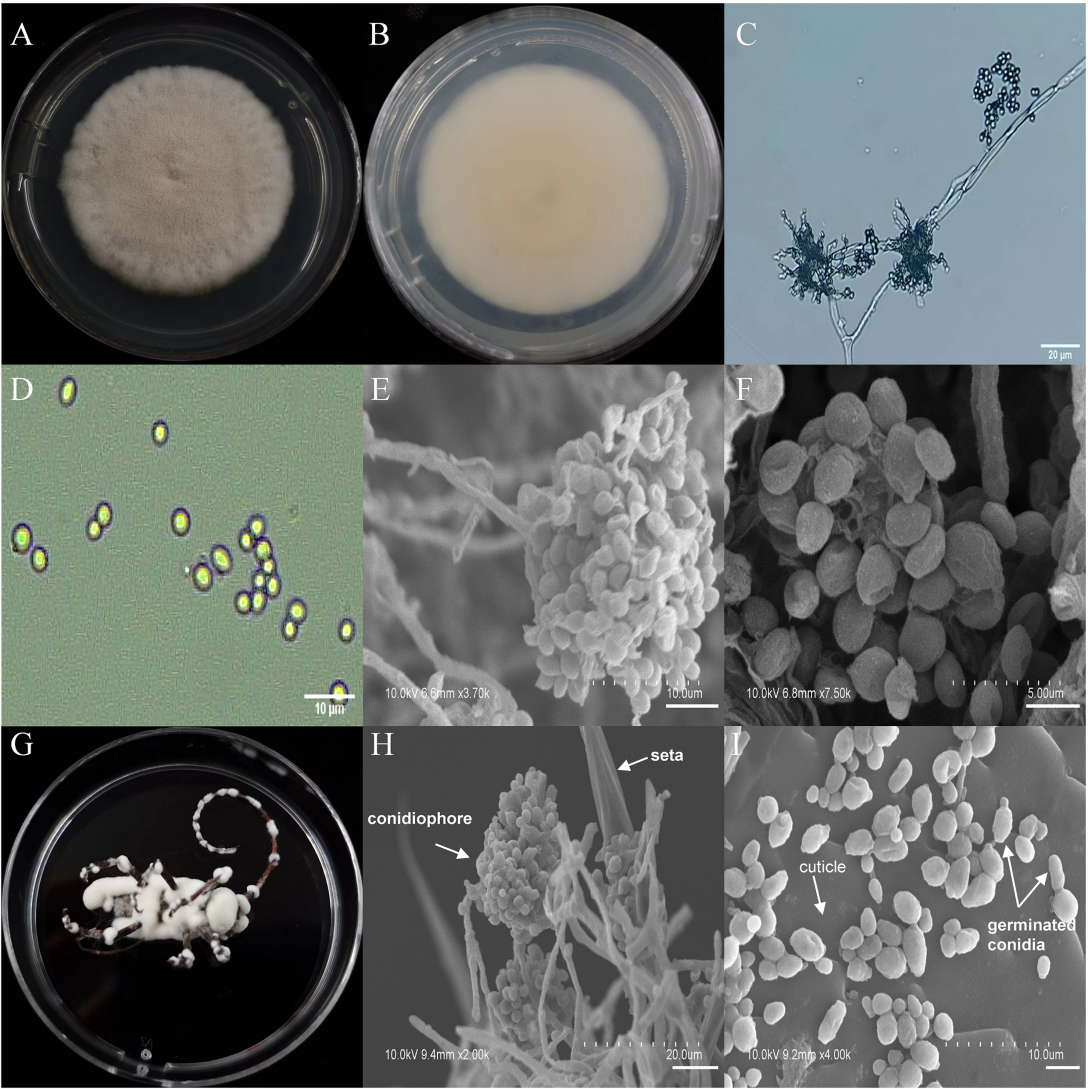
Morphology of *B*. *bassiana* and *M*. *alternatus* cadaver infected by *B*. *bassiana* under optical microscope and SEM. **(A)** Colonial morphology cultured on PDA. **(B)** Reverse of colony on PDA. **(C)** Hyphae and conidiophores (OM). **(D)** Conidia (OM). **(E)** Conidiophores (SEM). **(F)** Conidia (SEM). **(G)** *M*. *alternatus* cadaver surrounded by mycelia. **(H)** Conidiophores grown from *M*. *alternatus* cuticle (SEM). **(I)** Conidia on *M*. *alternatus* cuticle surface (SEM).

**Figure 5:**
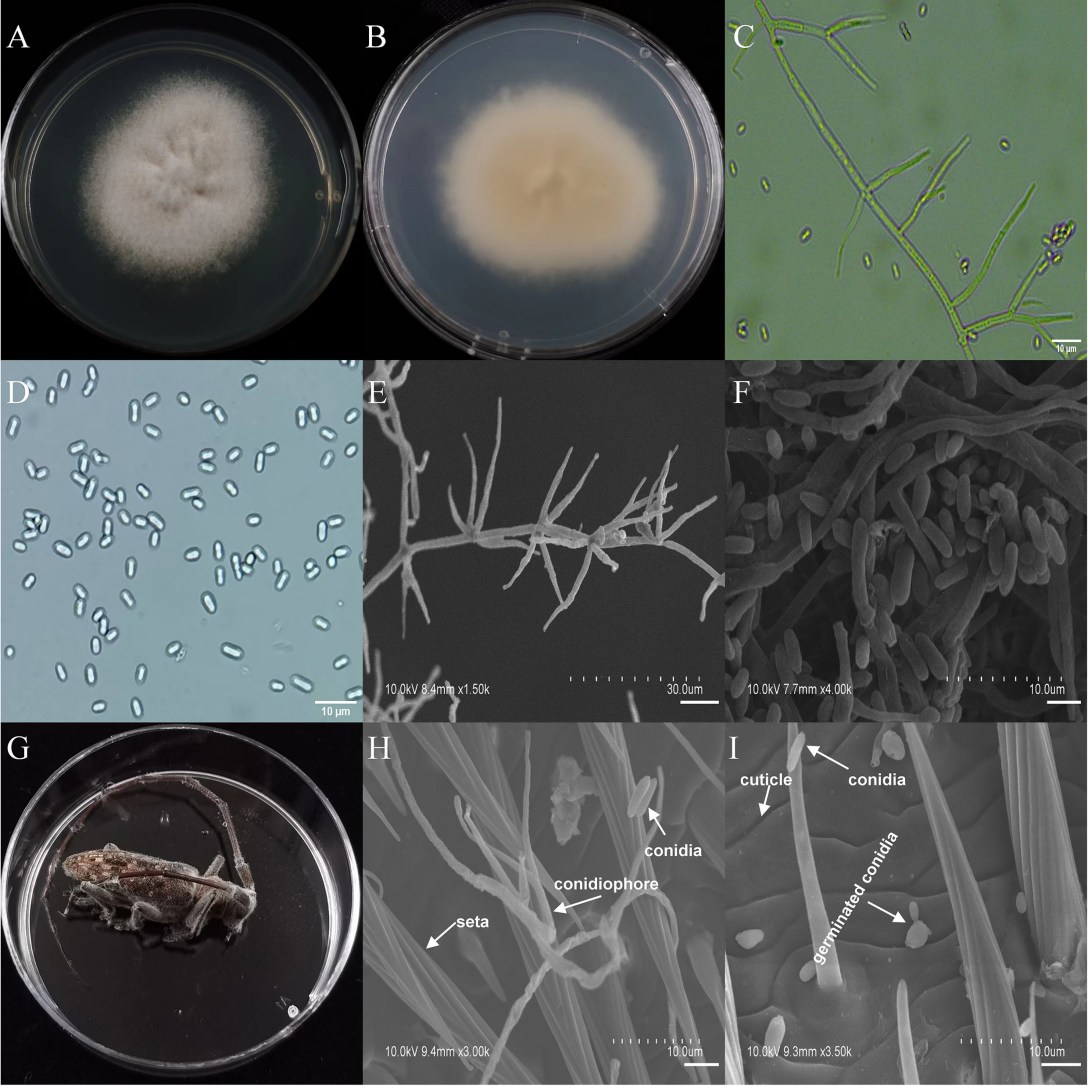
Morphology of *L*. *attenuatum* and *M*. *alternatus* cadaver infected by *L*. *attenuatum* under optical microscope and SEM. **(A)** Colonial morphology cultured on PDA. **(B)** Reverse of colony on PDA. **(C)** Hyphae and conidiophores (OM). **(D)** Conidia (OM). **(E)** Conidiophores (SEM). **(F)** Conidia (SEM). **(G)** *M*. *alternatus* cadaver surrounded by mycelia. **(H)** Conidiophores grown from *M*. *alternatus* cuticle (SEM). **(I)** Conidia on *M*. *alternatus* cuticle surface (SEM).

**Figure 6:**
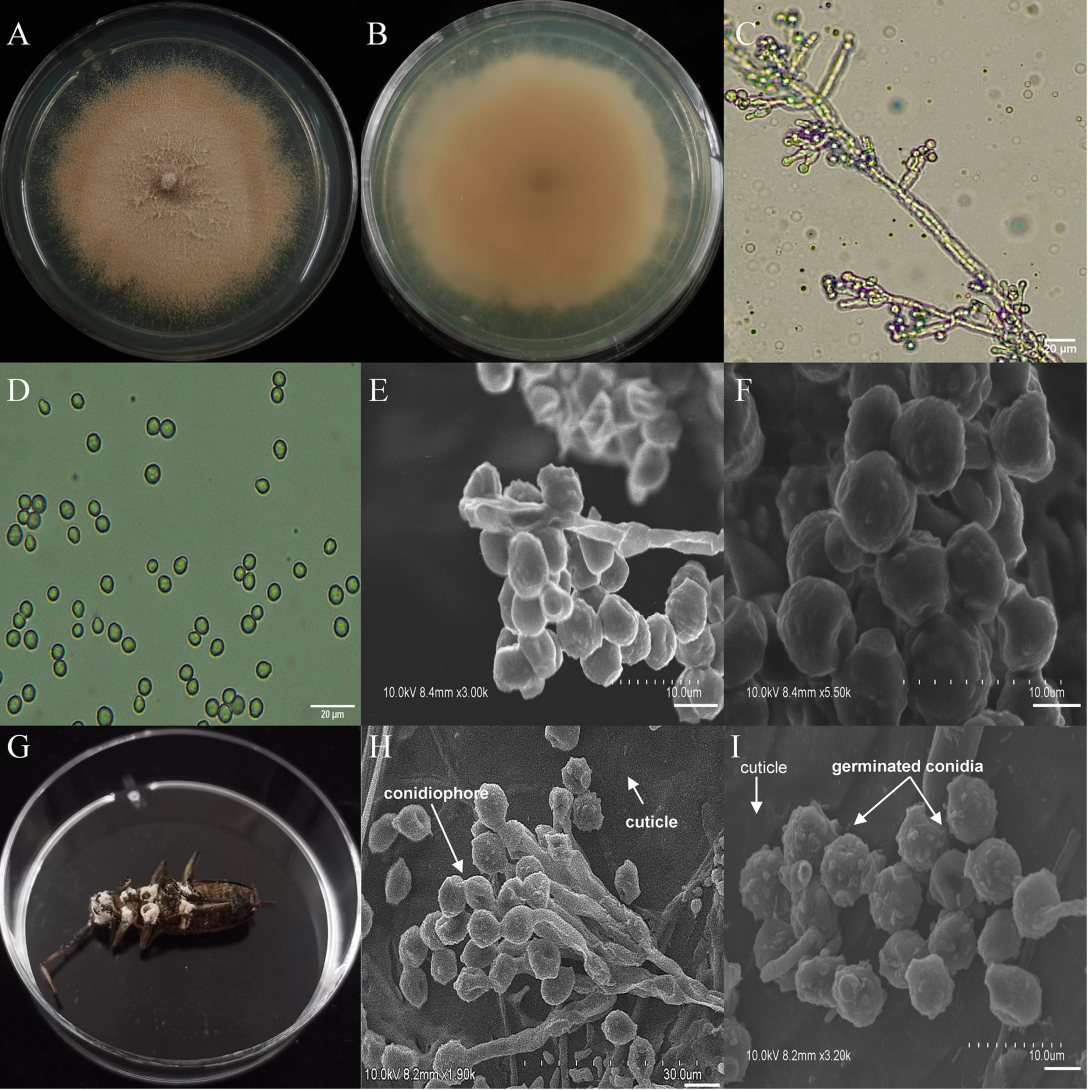
Morphology of *S*. *alboflavescens* and *M*. *alternatus* cadaver infected by *S*. *alboflavescens* under optical microscope and SEM. **(A)** Colonial morphology cultured on PDA. **(B)** Reverse of colony on PDA. **(C)** Hyphae and conidiophores (OM). **(D)** Conidia (OM). **(E)** Conidiophores (SEM). **(F)** Conidia (SEM). **(G)** *M*. *alternatus* cadaver surrounded by mycelia. **(H)** Conidiophores grown from *M*. *alternatus* cuticle (SEM). **(I)** Conidia on *M*. *alternatus* cuticle surface (SEM).

Among the seven entomopathogenic fungi that caused significant fitness loss in *T*. *castaneum* adults, three fungi (*A*. *ruber*, *P*. *citrinum*, and *T*. *dorotheae*; grown on PDA medium; **Figures S6–S8**) did not display visible infection phenotypes on the beetle, neither did they on *M*. *alternatus*. However, the other four (*A*. *austwickii*, *B*. *bassiana*, *L*. *attenuatum*, and *S*. *alboflavescens*) showed conspicuous infection symptoms on *T*. *castaneum* body surface (**Figure S9**), indicating strong parasitic capacities of these fungi. Their infection phenotypes were also observed in *M*. *alternatus* adult bodies, with mycelia of the four fungal species penetrating the body surface from the inside of the beetle, carrying asexual conidiophores (**Figures 3**–**6**).

The asexual morphological features of parasitic entomopathogenic fungi grown on PDA medium and the bodies of the two beetle species were observed and as follows: *A*. *austwickii* colony was initially white, and then became dark green due to the production of spores (**Figure 3A**). The reverse color was brown and green (**Figure 3B**). The conidiophores were smooth-walled and contained pyriform-shaped vesicles (**Figure 3C**, by optical microscope, OM). The conidia were globose, 3.5–4.4 μm×3.1–3.6 μm in diameter (**Figure 3D**, by OM) and grown from ampulliform phialides on the conidiophore (**Figure 3E**, by SEM). The conidia were shown with rough walls (**Figure 3F**, by SEM). *M*. *alternatus* cadaver was covered with yellowish green mycelia of *A*. *austwickii* (**Figure 3G**; mainly on its abdomen). Conidiophores grown from *M*. *alternatus* (**Figure 3H**, by SEM) and *T*. *castaneum* beetles (**Figure S9A**) had radial conidial heads typical of the Genus *Aspergillus*, producing spherical conidia with rough and echinulate walls (**Figure 3I**, by SEM; **Figure S9B**). *B*. *bassiana* formed a downy white colony with a powdery texture (**Figure 4A**), and the reverse was milky white (**Figure 4B**). Hyphae were septate and branched (**Figure 4C**, by OM) and the subglobose conidia were 2.9–3.4 μm×1.7–2.1 μm in diameter (**Figure 4D**, by OM; **Figure 4F**, by SEM). Conidiophores consisted of dense and spherical lateral clusters of globose to flask-shaped conidiogenous cells on top of an elongating and geniculate rachis (**Figure 4C**, by OM; **Figure 4E**, by SEM). *M*. *alternatus* cadaver was wholly covered by *B*. *bassiana* mycelia (**Figure 4G**), and the characteristics of conidiophores (**Figure 4H**, by SEM) and conidia (**Figure 4I**, by SEM) on the beetle were the same as those on PDA medium and *T*. *castaneum* (**Figures S9C, D**). *L*. *attenuatum* colony was white and cottony (**Figure 5A**), and the reverse side was light yellow in the center with a white margin (**Figure 5B**). Hyphae were smooth-walled, carrying conidiophores in solitary, opposite, or verticillate, with long and sharp-tipped phialides that sometimes branched (**Figure 5C**, by OM; **Figure 5E**; by SEM). The conidia were cylindrical with slightly narrowed ends or elliptical sharp, and they were 3.2–4.4 μm×1.6–1.9 μm in diameter (**Figure 5D**, by OM; **Figure 5F**, by SEM). *M*. *alternatus* cadaver was covered with a thin layer of mycelia, especially on its head and antennae (**Figure 5G**). The conidiophores with thorn-like phialides were grown from infecting mycelia on *M*. *alternatus* (**Figure 5H**, by SEM) and *T*. *castaneum* (**Figure S9E**), and cylindrical to oval conidia were found attached to the cuticles of the two beetle species (**Figure 5I**, by SEM; **Figure S9F**). *S*. *alboflavescens* initially formed a powdery and translucent colony, which then turned yellowish brown with an irregular margin (**Figure 6A**). The reverse of the colony had a pale yellowish margin around the tawny center (**Figure 6B**). Cylindrical to slightly flask-shaped phialides were clustered or occasionally grown solitarily on the main stem or branches of the conidiophores (**Figure 6C**, by OM). The conidia were globose to subglobose, 6.0–7.9 μm×5.6–6.9 μm in diameter (**Figure 6D**, by OM), and arranged in chain (**Figure 6E**, by SEM) with truncate base (**Figure 6F**, by SEM). The mouthparts and leg base nodes of *M*. *alternatus* cadaver were covered with pale yellowish mycelia (**Figure 6G**). Clustered conidiophores with long and slim phialides were grown from *M*. *alternatus* (**Figure 6H**, by SEM) and *T*. *castaneum* (**Figure S9G**) bodies. Conidia on beetle cuticles were rough-walled, having a truncate base (**Figure 6I**, by SEM; **Figure S9H**), similar to those on PDA medium.

In the ML phylogenetic analysis, the six-gene sequences of 65 species (including the seven entomopathogenic fungal species isolated in this study) were used to reconstruct the phylogenetic framework (**Table S1**; **Figure S10**). *A*. *austwickii* strain HUZU6 and *A*. *ruber* strain HUZU28 were clustered with their respective reference strains and were separated from other *Aspergillus* species. *P*. *citrinum* strain HUZU144 clustered into the clade of *P*. *citrinum* CBS 139.45, was distinct from the other *Penicillium* species (**Figure S10**). *S*. *alboflavescens* strain HUZU190 clustered well into a clade with *S*. *alboflavescens* CBS 399.34, which was distinct from the other *Scopulariopsis* species (**Figure S10**). *B*. *bassiana* strain HUZU62, *L*. *attenuatum* strain HUZU100, and *T*. *dorotheae* strain HUZU218 matched to their corresponding reference strains and were less distinct from their phylogenetically related species (**Figure S10**). The results of the multi-gene phylogenetic analysis conformed to the morphological features of the entomopathogenic fungal species.

### Insect-parasitic entomopathogenic fungi are lower in phytopathogenicity to the host pine, *P*. *massoniana*

**Figure 7:**
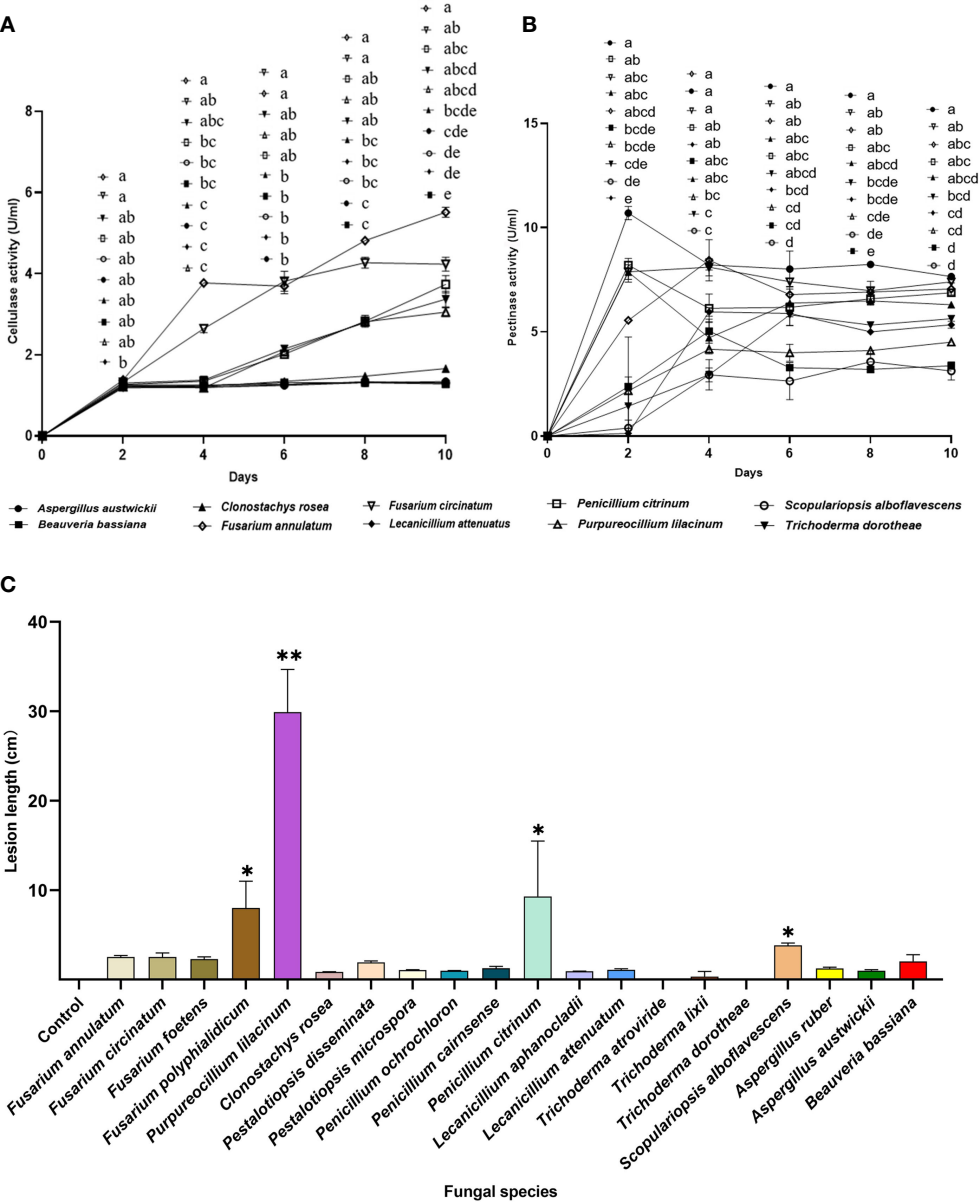
Phytopathogenic activities of the entomopathogenic fungi to the host pine *P*. *massoniana.* **(A)** Cellulase activities of fermentation supernatants from fungal species. **(B)** Pectinase activity of fermentation supernatants from fungal species. **(C)** Lesion lengths caused by fungi after 3 weeks. In A and B, different letters mean significant differences among fungi at each time point (*P<* 0.05); In C, asterisks on bars mean significant difference between the control group and fungal species (* *P<* 0.05; ** *P<* 0.01). Data were represented as Mean ± SD.

*Fusarium* is an important Genus of pine pathogenic fungi with a relatively high isolation frequency in *M*. *alternatus*. Therefore, *Fusarium* species were included as positive controls for entomopathogenic fungi, to evaluate their potential ability to damage the host pine *P*. *massoniana*. Five fungal species, namely, *F*. *annulatum*, *F*. *circinatum*, *P*. *citrinum*, *P*. *lilacinum*, and *T*. *dorotheae*, showed dramatic increases in cellulase activity levels with incubation time. In contrast, cellulase activity levels of the insect-parasitic entomopathogens *A*. *austwickii*, *B*. *bassiana*, *L*. *attenuatum* and *S*. *alboflavescens* did not increase over time and were lower than those of the above five fungal species (**Figure 7A**). Regarding pectinase activity, all fungal species elevated their activity levels and remained stable at the end of the incubation, although these species showed variable time points to reach their peak activity (**Figure 7B**). *F*. *annulatum*, *F*. *circinatum*, and *P*. *citrinum* exhibited significantly higher pectinase activity than *B*. *bassiana* and *S*. *alboflavescens*, on days 8 and 10. With the exception of *A*. *austwickii*, which expressed the highest level of pectinase, the other three insect-parasitic entomopathogens showed almost the lowest pectinase activity among all fungi (**Figure 7B**).

Lesion lengths caused by fungal associates on *P*. *massoniana* seedlings further demonstrated lower pine pathogenicity of insect-parasitic entomopathogenic fungi than the other fungal species (**Figure 7C**; Kruskal-Wallis test; χ_20_^2^ = 59.80, *P*< 0.0001). One insect-parasitic entomopathogenic fungus, *S*. *alboflavescens*, slightly induced necrosis in the cambial zone of host pine, while exhibiting the lowest cellulase and pectinase activities among all fungi. *A*. *austwickii* had high pectinase activity but did not cause obvious lesions on the pine. Some fungal associates isolated from *M*. *alternatus*, such as *F*. *polyphialidicum* in Genus *Fusarium*, *P*. *lilacinum*, and *P*. *citrinum*, induced significantly greater lesions than the mock inoculation control (**Figure 7C**).

## Discussion

In this study, the pine sawyer beetle *M*. *alternatus* of naturally fungal infection is associated with a strikingly high diversity of fungal communities, with 640 fungal strains in total affiliated to 7 orders, 13 families, 15 genera, and 39 species (**Table 1**). This is distinct from previously considered, which held that entomopathogenic fungal species of *M*. *alternatus* are restricted to genera mainly in *Beauveria* and *Metarhizium* (Kim et al., 2020; Kim et al., 2022). *B*. *bassiana* was reported as the dominant entomopathogenic fungus of *M*. *alternatus* in Japan, Korea, and the Anhui/Zhejiang Province of China (Shimazu, 2004; Han et al., 2007; Ma et al., 2009; Shin et al., 2009), and *B*. *pseudobassiana*, which is closely related to it, was the most frequently isolated species from infected adults of *M*. *galloprovincialis* in Spain (Álvarez-Baz et al., 2015), implying *Beauveria* spp. to be the main infecting fungi of *Monochamus* vector beetles in natural fields. However, in this survey, only two strains of *B*. *bassiana* were isolated from the body surface of *M*. *alternatus* adults in the Zhejiang population, and no strains were found in the other populations. *Lecanicillium* species are pathogenic parasites to various insect species (Goettel et al., 2008). In *Monochamus* insect pests, *L*. *lecanii* (formerly named *Verticillium lecanii*) was reported to associate with naturally infected *M*. *alternatus* in Anhui/Zhejiang regions of China (Han et al., 2007; Ma et al., 2009). Its closely related species, *L*. *attenuatum*, was found to accompany *B*. *pseudobassiana* in *M*. *galloprovincialis* populations in Spain (Álvarez-Baz et al., 2015), and to the best of our knowledge, our study is the first report to elucidate such an association of *L*. *attenuatum* with *Monochamus* spp. in China. It is highly possible that the identity of *L*. *lecanii* determined by morphological traits is that of *L*. *attenuatum* in the Zhejiang population of *M*. *alternatus*, which needs to be further confirmed. *Scopulariopsis* is commonly found in various habitats, and some species, such as *S*. *asperula* and *S*. *brevicaulis*, have been isolated from mites and insects with entomopathogenic activities (Perrucci et al., 2008; Woudenberg et al., 2017). The species *S*. *alboflavescens* isolated from *M*. *alternatus* in this survey was reported to be hosted only by mammals as a pathogenic fungus (Woudenberg et al., 2017; Pérez-Cantero and Guarro, 2020), with no record on insects. The two dominant fungal genera *Aspergillus* and *Penicillium* of *M*. *alternatus* found in this study are eminent producers of secondary metabolites with diverse structures, many possessing excellent insecticidal properties that target insect metabolic systems (Frisvad et al., 2018; Toghueo and Boyom, 2020). Representative species such as *A*. *ruber*, *A*. *austwickii*, and *P*. *citrinum* among the collected isolates formed the first report of their associations with *Monochamus* spp., as per published record.

Entomopathogenic fungi present a geographical distribution preference for specific *M*. *alternatus* populations. Results of this field investigation revealed that the fungal species with insecticidal activities, namely, *L*. *attenuatum* (50.8%), *A*. *austwickii* (41.7%), *S*. *alboflavescens* (31.4%), and *A*. *ruber* (85.1%), were mainly found or dominant in Zhejiang, Sichuan, Fujian, and Guangxi, respectively, in *M*. *alternatus* populations (**Table S2**). This implied that variation in optimal growth ranges of abiotic factors, such as temperature and humidity, might directly determine their distributions. Multivariate analysis confirmed a significant geographical distribution pattern in the community composition of fungal associates in naturally infected *M*. *alternatus* (**Figure 1**). More significant differences in fungal community composition were found in geographically distant *M*. *alternatus* populations. Latitudinal geographies with distinct environmental factors, such as climate and vegetation, could shape the diversity and composition of many types of fungal communities (Větrovský et al., 2019; Mukhtar et al., 2021), including insect-associated fungi (such as ophiostomatoid fungal symbionts with bark beetles) (Roe et al., 2011). Referring to the potential distribution preferences of entomopathogenic fungi, certain species or their phylogenetically close species matched well with the high frequencies at which they were isolated and the environmental conditions for their optimal growth. For example, *L*. *attenuatum* was isolated from soil in Korea (Woo et al., 2020) and from *M*. *galloprovincialis* in Spain, which are located at high latitudes. *L*. *flavidum*, its congeneric species, was demonstrated to have a narrow growth temperature range from 18°C to 21°C, with no growth at 27°C (Zare and Gams, 2008). In contrast, the growth temperatures of *Aspergillus* and *Scopulariopsis* appear to be much higher than those of *Lecanicillium* species. Although *Aspergillus* can grow across a broad temperature range, many species of this Genus occur more frequently at tropical latitudes, with growth at temperatures from 30°C to 37°C (Klich, 2002; Belli et al., 2004). Another study pointed out that the optimal growth temperature of *S*. *brevicaulis* is 30°C, which is also optimal for enzyme production (Anbu et al., 2007). The vector beetle *M*. *alternatus* has a wide range of potential distribution areas in China, providing diverse reservoirs of natural resources for exploring original entomopathogenic fungal strains with huge economic value. As shown in this study, more entomopathogenic fungi will be found in *M*. *alternatus* through deeper field investigation and developed as novel biopesticides to control pine wilt disease in the future.

The behavioral phenotypes of the seven fungi with strong insecticidal activities against *T*. *castaneum* adults were consistent with those against *M*. *alternatus*. The four parasitic entomopathogenic fungi, namely, *A*. *austwickii*, *B*. *bassiana*, *L*. *attenuatum*, and *S*. *alboflavescens*, could be re-isolated from *T*. *castaneum* with artificial infection in the laboratory, and the SEM results confirmed that the asexual characteristics of conidia and conidiophores grown on *T*. *castaneum* were the same as those grown on *M*. *alternatus* (**Figure S9**; **Figures 3**–**6**). Mycelia of these four fungal species grew intensively on the abdomen, whole body, head and antenna, and mouthpart and leg base nodes of adult *M*. *alternatus*, respectively (**Figures 3G**, **4G**, **5G**, **6G**), suggesting that these fungal species have spatial preference and localized niches on host insect bodies. However, multivariate analysis showed that the fungal community composition did not diverge significantly among different body positions, although there was a slight variation among the groups (**Figure S3**). The other three insecticidal fungal species with no visible parasitism on *M*. *alternatus*, namely, *A*. *ruber*, *P*. *citrinum*, and *T*. *dorotheae*, failed re-isolation from artificially infected *T*. *castaneum* and also were not observed by SEM. *T*. *castaneum* is a Coleopteran model insect widely used in genetic editing, toxicological and immunological research, and active natural product screening (Wang et al., 2007; Klingler and Bucher, 2022), owing to its advantages of short generation-time and simple rearing techniques (Rösner et al., 2020). The consistent results found here further indicate that the laboratory population of *T*. *castaneum* could be a good substitute or a tool to evaluate insecticidal activities and infection phenotypes of entomopathogenic fungi for *M*. *alternatus* and other Coleopterans.

Entomopathogenic fungi harbor diverse biological “weapons” and apply several strategies to kill target insect hosts, including enzymatic degradation, physical penetration of integuments, propagation within host body cavity (hemocoel), and/or mycotoxin excretion (Mannino et al., 2019; Litwin et al., 2020). For parasitic entomopathogenic fungi, the first step to colonize host body surface relies on the efficient release of protease, chitinase, and lipase to degrade cuticles (Sevim et al., 2012; Umaru and Simarani, 2022), and these three extracellular enzymes are direct indicators of their insecticidal activity (Mustafa and Kaur, 2009; Grewal et al., 2021). In this study, *A*. *austwickii* isolated from *M*. *alternatus* showed moderate chitinase activity while higher protease and lipase activities, which correlated with its obvious infection phenotype and higher mortality rate, compared to other species (**Figures 2**, **3**). Similar result was found in *B*. *bassiana*, which expressed particularly high level of protease, causing high mortality of the test beetle *T*. *castaneum* (**Figures 2**, **4**). This conformed with a previous study, which found that mortality rates of *M*. *alternatus* positively correlated with protease activity levels of *B*. *bassiana* (Lin et al., 2008). *L*. *attenuatum* presented significant parasitism on *M*. *alternatus* (**Figure 5**), analogous to *A*. *austwickii* and *B*. *bassiana*, while the performance of *L*. *attenuatum*—in terms of the three enzymatic activities—was lower than that of the other two species, which could explain the weaker virulence of *L*. *attenuatum* on the test beetle (**Figure 2**) as well as on *M*. *galloprovincialis* adults and *Oryctes agamemnon* larvae (Álvarez-Baz et al., 2015; Saleem and Ibrahim, 2019). *S*. *alboflavescens* expressed relatively high level of chitinase activity, but its low protease and lipase productivities were not equivalent to its marked infection phenotype and strong pathogenicity to beetles (**Figures 2**, **6**). Determination of the exact roles of enzymes involved in *S*. *alboflavescens* pathogenesis requires further investigation. Fungal secretion of toxic secondary metabolites could accelerate the death of insect hosts alongside enzyme-initiating infection. The chemical action might even be the principal mode for non-parasitic entomopathogenic fungi, such as *Trichoderma* species, which were reported to capable of producing insecticidal secondary metabolites, antifeedant compounds, and repellent chemicals (Poveda, 2021). The structural nature of these secondary metabolites is worthy of exploring for their efficacies against *M*. *alternatus* in the future.

Interactions between insect hosts and entomopathogenic fungi exhibit host specificity and strain-level variation in certain fungal species. For example, in this study, the *P*. *lilacinum* strain isolated from *M*. *alternatus* expressed low-to-moderate level of protease (**Figure 2B**), but no obvious parasitism or mortality was found when interacting with the test beetles (**Figure 2A**). However, *P*. *lilacinum* has been reported to be a strong parasitic entomopathogen of aphid and moth insect pests that secretes proteases and chitinases (Liu et al., 2022). Similarly, *P*. *disseminata* and *C*. *rosea* did not show any insecticidal activity to the test beetles (**Figure 2A**), whereas they were natural parasites of the scale insect *Hemiberlesia pitysophila* and psyllid *Diaphorina citri via* enzymatic degradation of cuticles (Huang et al., 2014; Yang et al., 2021). Their low mortality in beetles and weak enzymatic behavior may serve the purpose of adhesion to the body surface without killing the host. The components and structure of cuticle of Coleopteran beetles are distinct from those of Hemipteran and other insect cuticles (Fraenkel and Rudall, 1947; Balabanidou et al., 2018), which could explain the variation of specific fungal species on different host insects. Strain-level genetic variation of entomopathogenic fungal species originating from different insect orders could also shape their specificity in host parasitism (Brodeur, 2012; Wang and Wang, 2017; Rohrlich et al., 2018), implying that the parasitic fungi on *M*. *alternatus* might fail to function on other distantly related hosts, and *vice versa*.

Many entomopathogenic fungal species have an endophytic lifestyle to provide chemical defense and growth promotion for host plants, and in return, gain benefits, such as space and nutrition from the plants (Jaber and Ownley, 2018; Bamisile et al., 2018; Bamisile et al., 2021). In this study, upon measuring levels of digestive enzymes that target plant cell wall components, *A*. *austwickii* consistently exhibited the highest level of pectinase activity, whereas the other three parasitic entomopathogenic fungi showed almost the lowest activity, which increased gradually with incubation time; however, all the four fungal species secreted tiny amounts of cellulase throughout the time (**Figures 7A, B**). In contrast, *T*. *dorotheae* and *P*. *citrinum* balanced well between the cellulase and pectinase activities (**Figures 7A, B**) and have been reported as endophytic fungi of other woody plants (Huh et al., 2011; Sadeghi et al., 2019). The phytopathogenicity assay showed that these entomopathogenic fungi are capable of growing in the host pine in a relatively mild manner (**Figure 7C**). This suggested that moderate expression of the two enzymes—under the threshold of pathogenesis—could facilitate the colonization of entomopathogenic fungi on the host pine *P*. *massoniana*, conferring them high compatibility with the host pine and meanwhile expanding their contact area with *M*. *alternatus* in the field. *P*. *citrinum* was an exception found in the assay, inducing much longer wounds in the host pine phloem (**Figure 7C**), which might be partially attributed to specific virulent factors of this fungus interactions with the pine immune system. Further study is needed to evaluate the non-host (especially natural enemy insects of *M*. *alternatus*) infection potential of the entomopathogenic fungi, which will provide more valuable views for their application prospect in biocontrol of the pine wilt disease.

In addition to transmitting the pine wood nematode, the adult beetle *M*. *alternatus* appears to be a multi-pathogen vector potentially carrying destructive pine-pathogenic fungi. *Fusarium* fungal species isolated from *M*. *alternatus* in this study showed extraordinarily high cellulase and pectinase production, and inoculation with *F*. *polyphialidicum* triggered a significant lesion on the host pine (**Figure 7**). *Fusarium* pathogenesis in pine forests, which leads to substantial economic losses worldwide, has attracted increased attention in recent years (Herron et al., 2015; Elvira-Recuenco et al., 2019). The survey in this study revealed that *Fusarium* spp. accounted for approximately 8% of all isolates from *M*. *alternatus* adults (**Table 1**), and the frequencies of association between *Fusarium* and *M*. *alternatus* or other *Monochamus* beetles have been recorded to be more than 30% (Han et al., 2007). Another fungal associate, *P*. *lilacinum*, caused much heavier damage to the *P*. *massoniana* phloem than the other fungi (**Figure 7C**), which is generally regarded as an effective protector of plants against various pest infestations (Lopez et al., 2014; Lopez and Sword, 2015). Its pathogenicity in the plantation system was observed only as a mushroom parasite (Masaphy, 2022). Nevertheless, considering the obvious symptom of *P*. *lilacinum* infection initially found in *P*. *massoniana*, its potential adverse effects on pine trees should be evaluated in future studies. These findings suggested a new underlying threat from fungal phytopathogens harbored by the vector *M*. *alternatus*, further highlighting the importance of developing novel fungal entomopathogenic bioagents for vector beetle management.

## Conclusion

Pine wilt disease, caused by the pinewood nematode (PWN), is a devastating vector-borne disease that has been infesting pine forests in several continents. Nevertheless, little attention has been paid to the diversity of entomopathogenic fungi naturally associated with vector beetles *Monochamus* spp. Our study initiated a preliminary investigation of entomopathogenic fungal associations in five geographical populations of the disease vector *M*. *alternatus* in southern China. This cross-latitudinal survey demonstrated significant variation in fungal community composition, coupled with the geographical origin of naturally infected *M*. *alternatus*. Furthermore, the main fungal species from the geographical populations were region-representative and strongly entomopathogenic, functioning in parasitic and non-parasitic modes. Among them, the natural distributions of insect-parasitic fungal species were discovered for the first time on *Monochamus* spp. in China, some of which showed potent insecticidal activities comparable to *B*. *bassiana*. These parasitic entomopathogenic fungi could hardly cause visible lesions on the host pine, which further supported their promising application in the field to break down vector transmission of pine wilt disease. Our findings reflect that cross-latitudinal resource exploration of entomopathogenic fungi is crucial for the development of novel biocontrol strategies for insect vector-borne diseases.

## Data availability statement

The original contributions presented in the study are included in the article and/or **Supplementary Material**. Further inquiries can be directed to the corresponding author.

## Author contributions

SW, LZ, and CC designed the research. CC supervised the experiments. SW, JW, YW, YQ, YH, JYW, and JC performed the experiments. SW and CC performed the bioinformatic and statistical analyses. SW and CC wrote the manuscript with all authors contributing to the discussion of the data. LZ and CC provided funds for the research. All authors contributed to the article and approved the submitted version.
