# Supplementary material for: Discovery of entomopathogenic fungi across geographical regions in southern China on pine sawyer beetle Monochamus alternatus and implication for multi-pathogen vectoring potential of this beetle
Source: Front Plant Sci. 2022 Dec 28;13:1061520. doi: 10.3389/fpls.2022.1061520 (PMC9832029; doi:10.3389/fpls.2022.1061520)
Supplement: Supplementary file 1 [file DataSheet_1.docx]

Supplementary Material

# Supplementary Figures and Tables

## Supplementary Figures

**
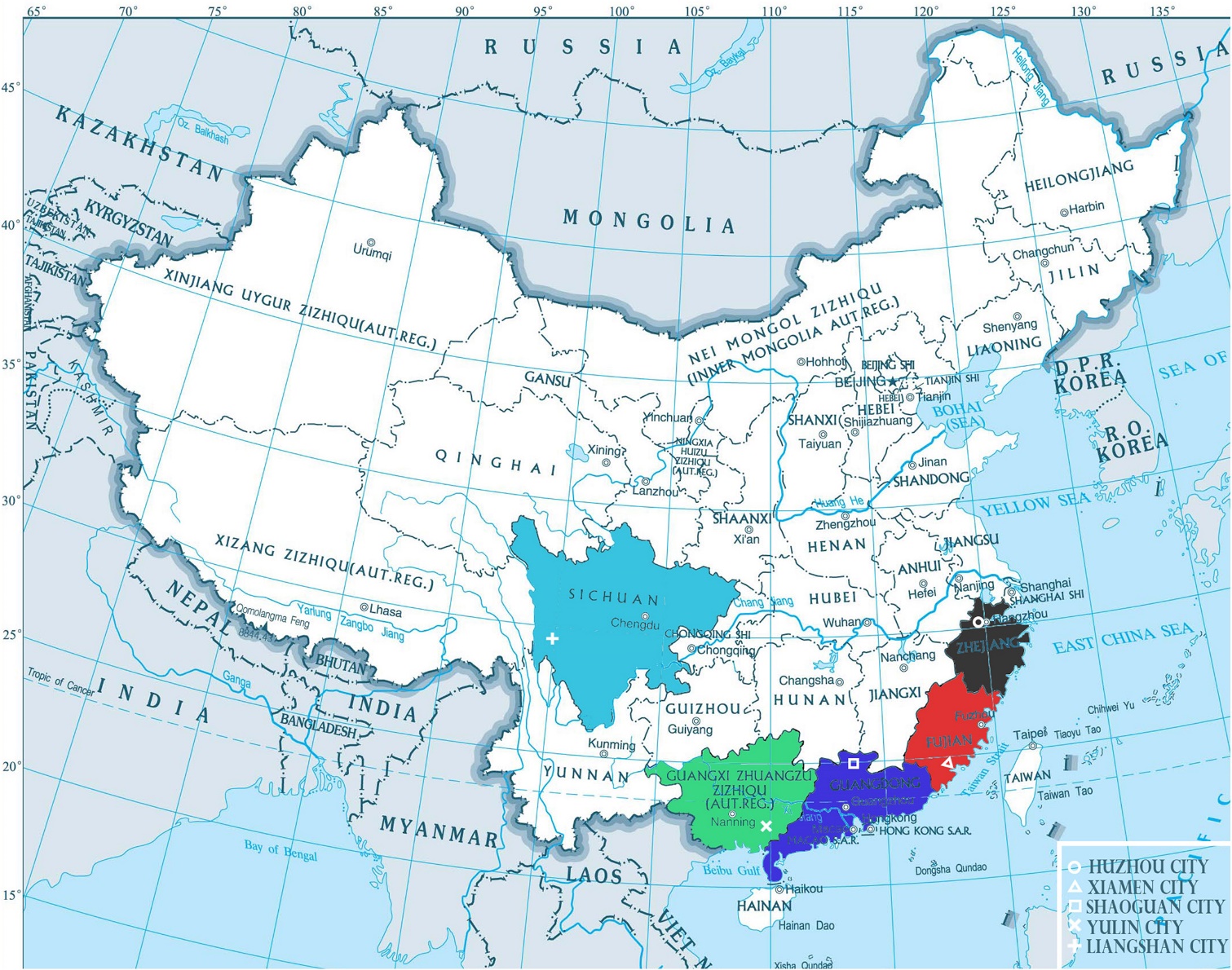
**

**Fig. S1.** Five geographical populations of *M*. *alternatus* in southern China. Different sampling regions were marked with different symbols and colors (Huzhou City in Zhejiang Province, Liangshan City in Sichuan Province, Xiamen City in Fujian Province, Shaoguan City in Guangdong Province and Yulin City in Guangxi Province).

**
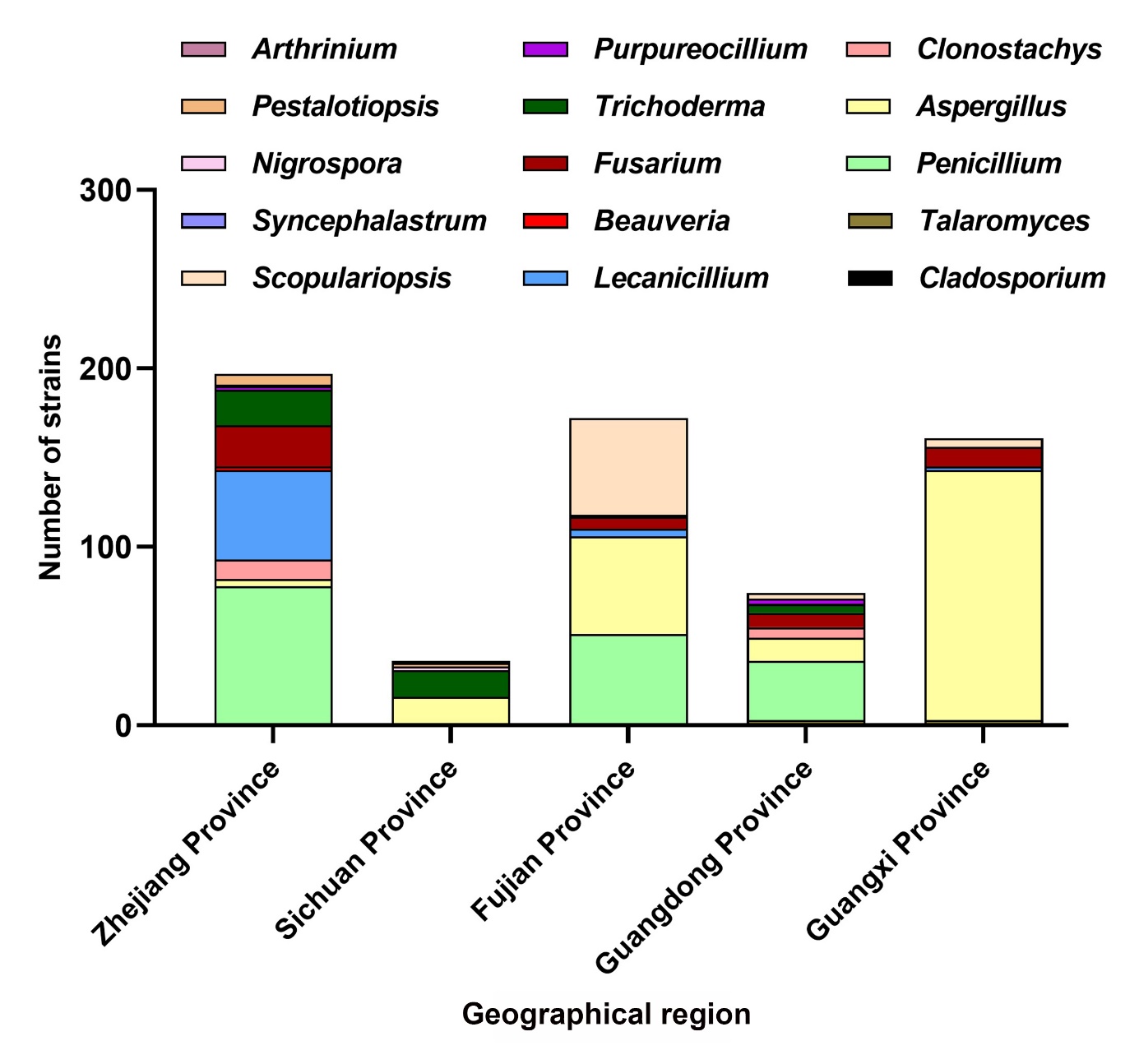
**

**Fig. S2.** The number of fungal strains belonging to each Genus isolated from different geographical populations of *M*. *alternatus*.


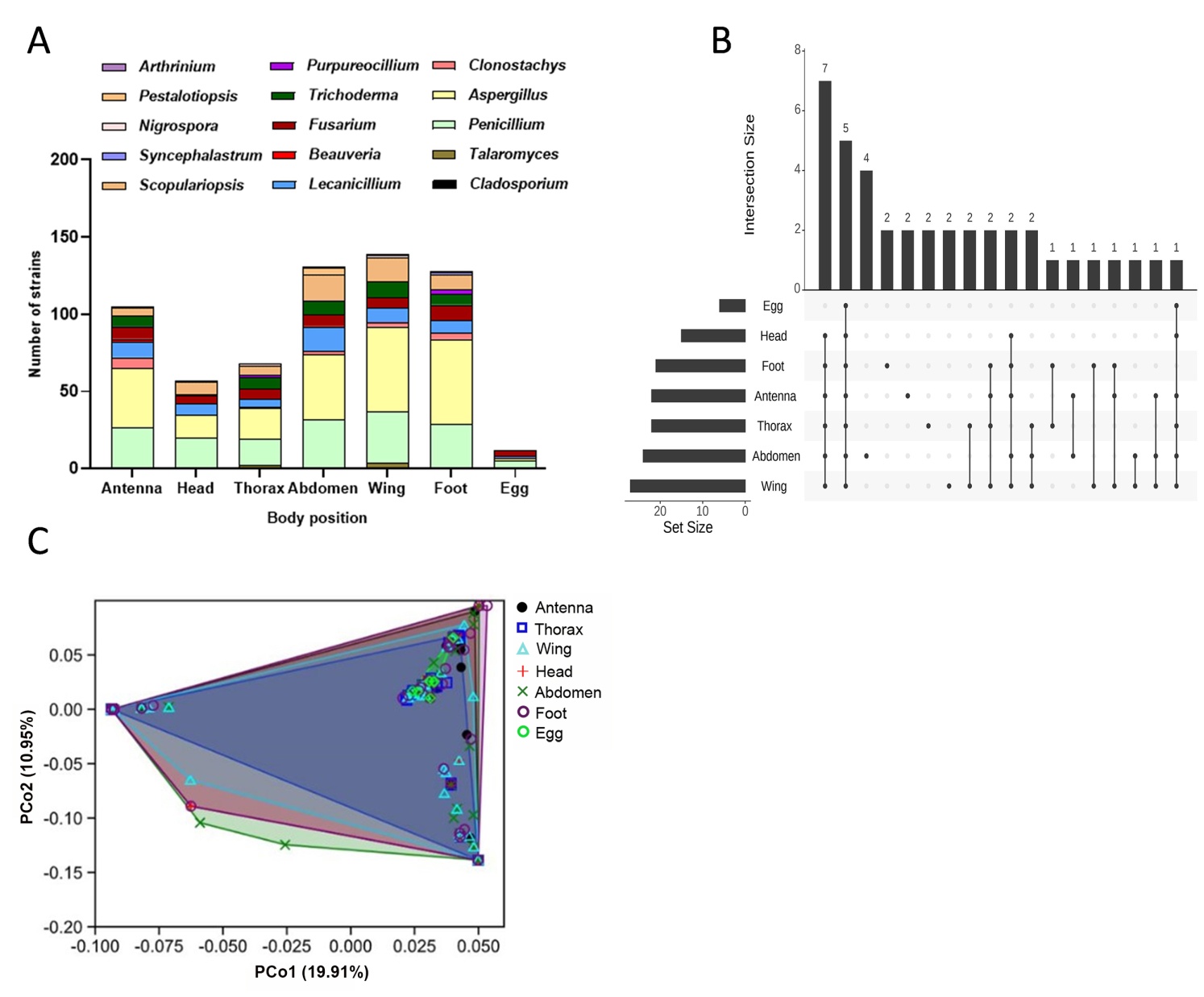


**Fig. S3.** Community composition variations of fungal associates of *M*. *alternatus* from different body positions. (A) The number of fungal strains belonging to each Genus isolated from different body position. (B) Upset plot. (C) PCoA of Bray-Curtis distance showing no obvious variation in fungal community composition among body positions.

**
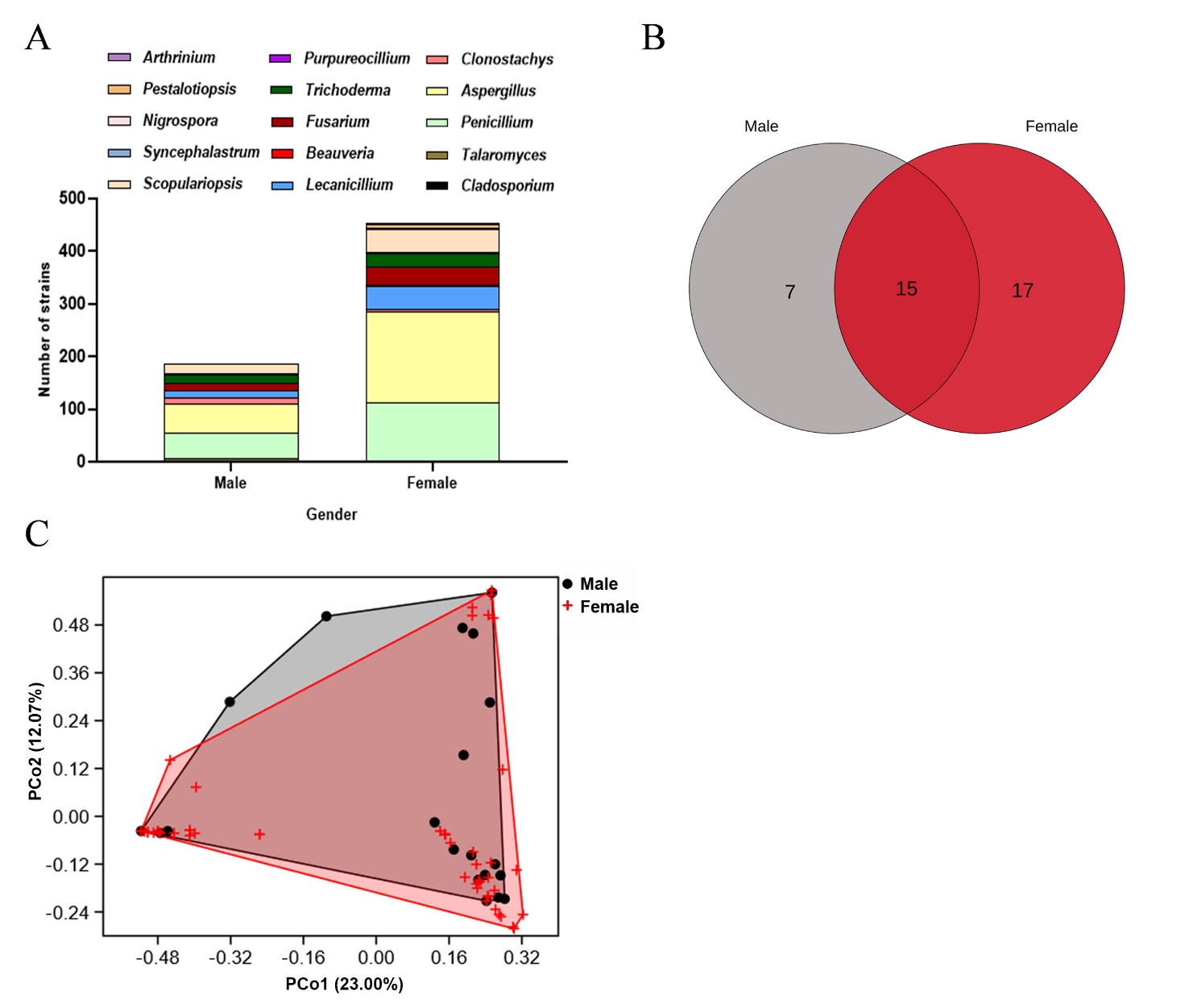
**

**Fig. S4.** Community composition variations of fungal associates of *M*. *alternatus* from different genders. (A) The number of fungi strains belonging to each Genus isolated from different genders. (B) Veen diagram. (C) PCoA of Bray-Curtis distance showing no obvious variation in fungal community composition between genders.

**
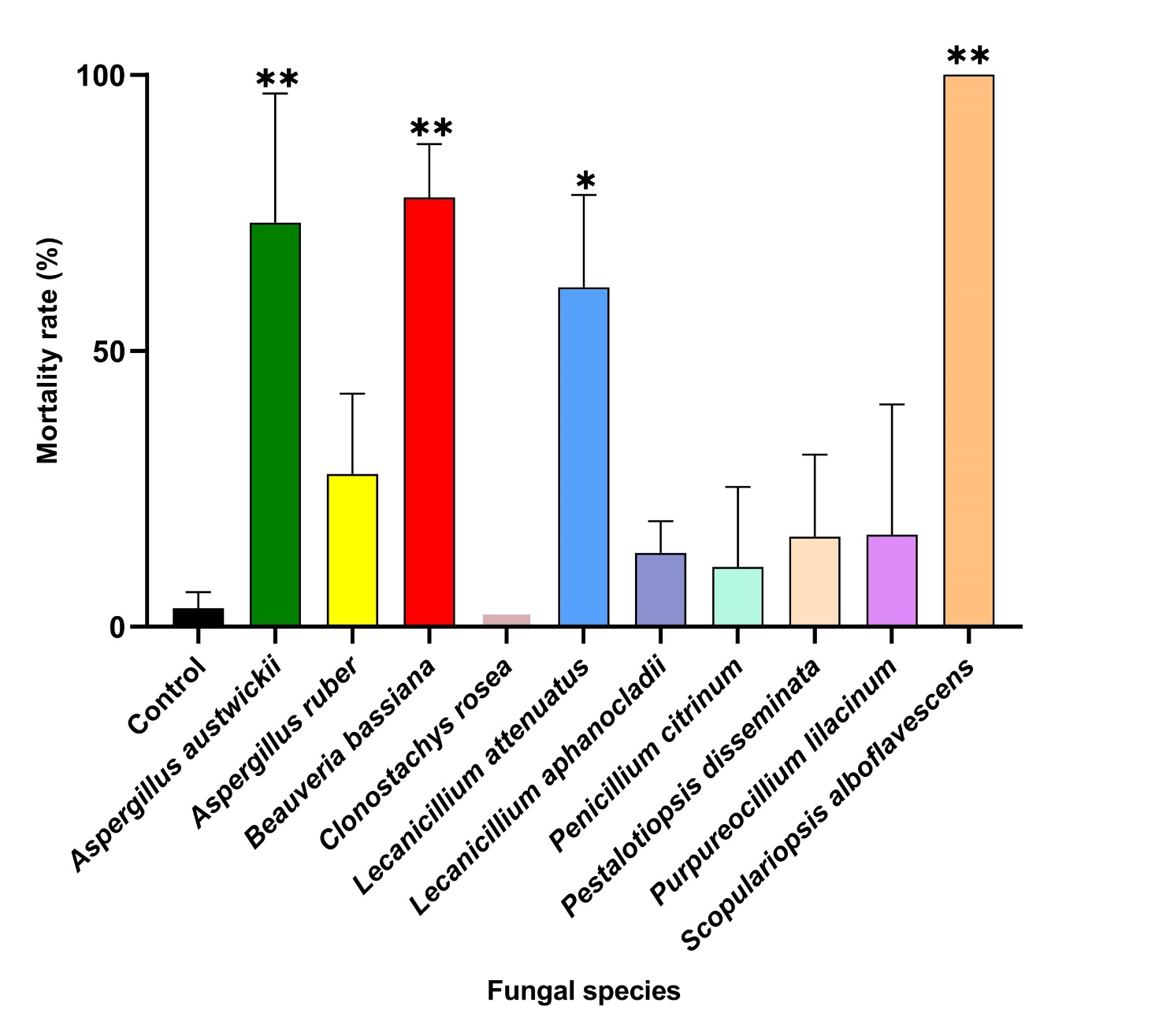
**

**Fig. S5.** Mortality rate of *Tribolium castaneum* beetles inoculated with the conidia suspension (1×10^8^ conidia/ml) of representative fungal species after 9 days. Asterisks on bars mean significant difference between the control group and fungal species (* *P* < 0.05; ** *P* < 0.01). Data were represented as Mean ± SD.

**
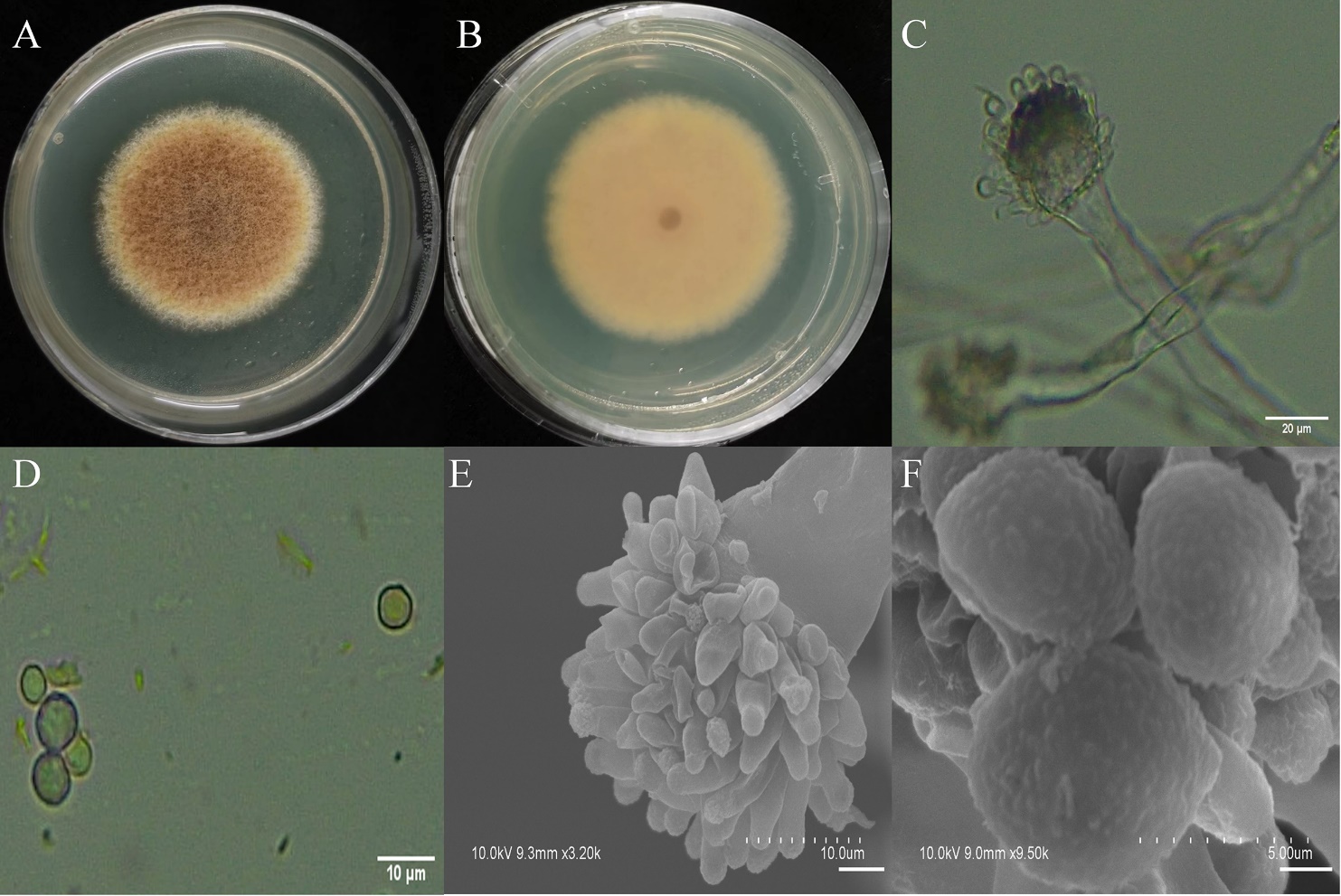
**

**Fig. S6.** Morphology of *A*. *ruber* under optical microscope and SEM. (A) Colonial morphology cultured on PDA. (B) Reverse of colony on PDA. (C) Hyphae and conidiophores (OM). (D) Conidia (OM). (E) Conidiophores (SEM). (F) Conidia (SEM).

**
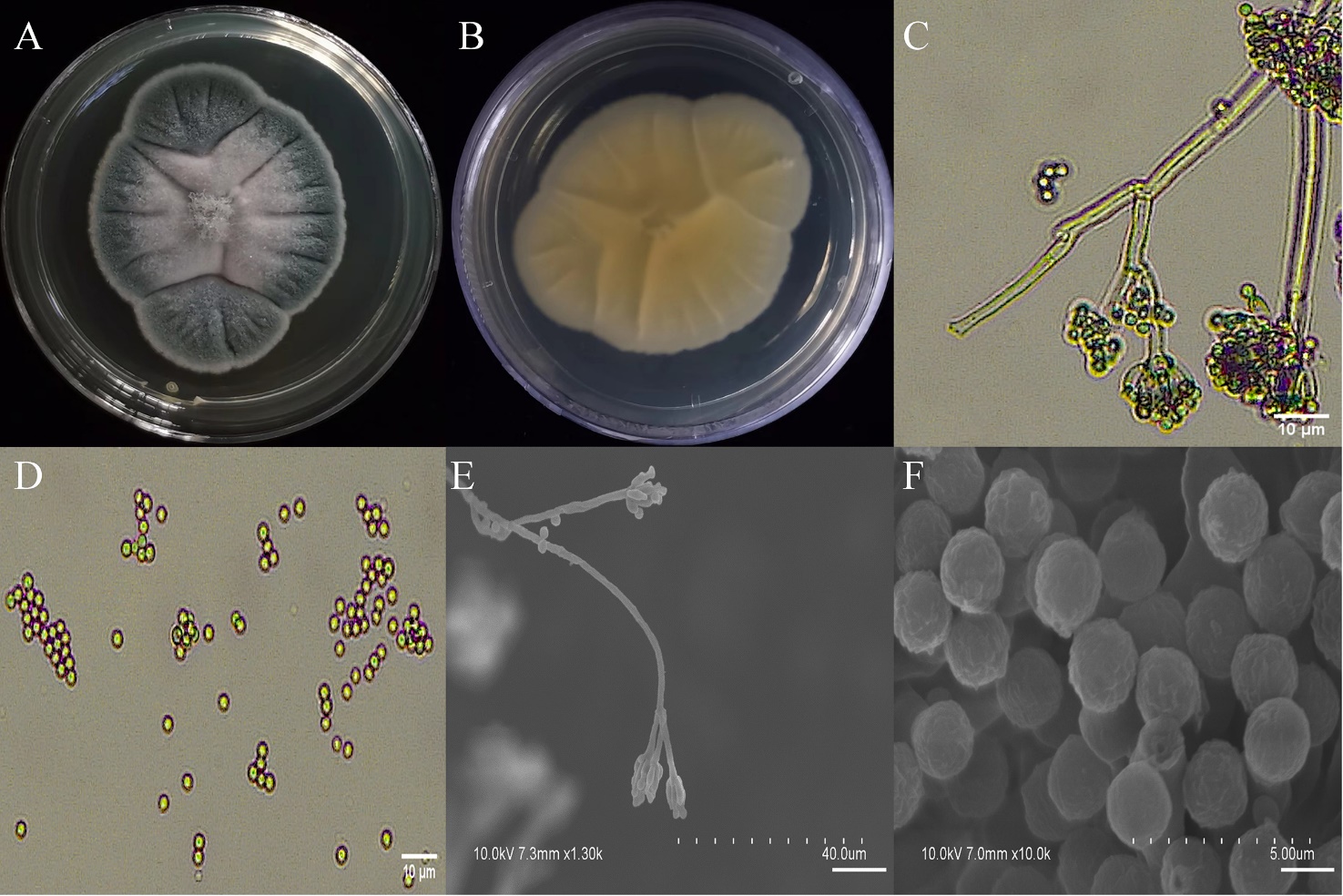
**

**Fig. S7.** Morphology of *P*. *citrinum* under optical microscope and SEM. (A) Colonial morphology cultured on PDA. (B) Reverse of colony on PDA. (C) Hyphae and conidiophores (OM). (D) Conidia (OM). (E) Conidiophores (SEM). (F) Conidia (SEM).

**
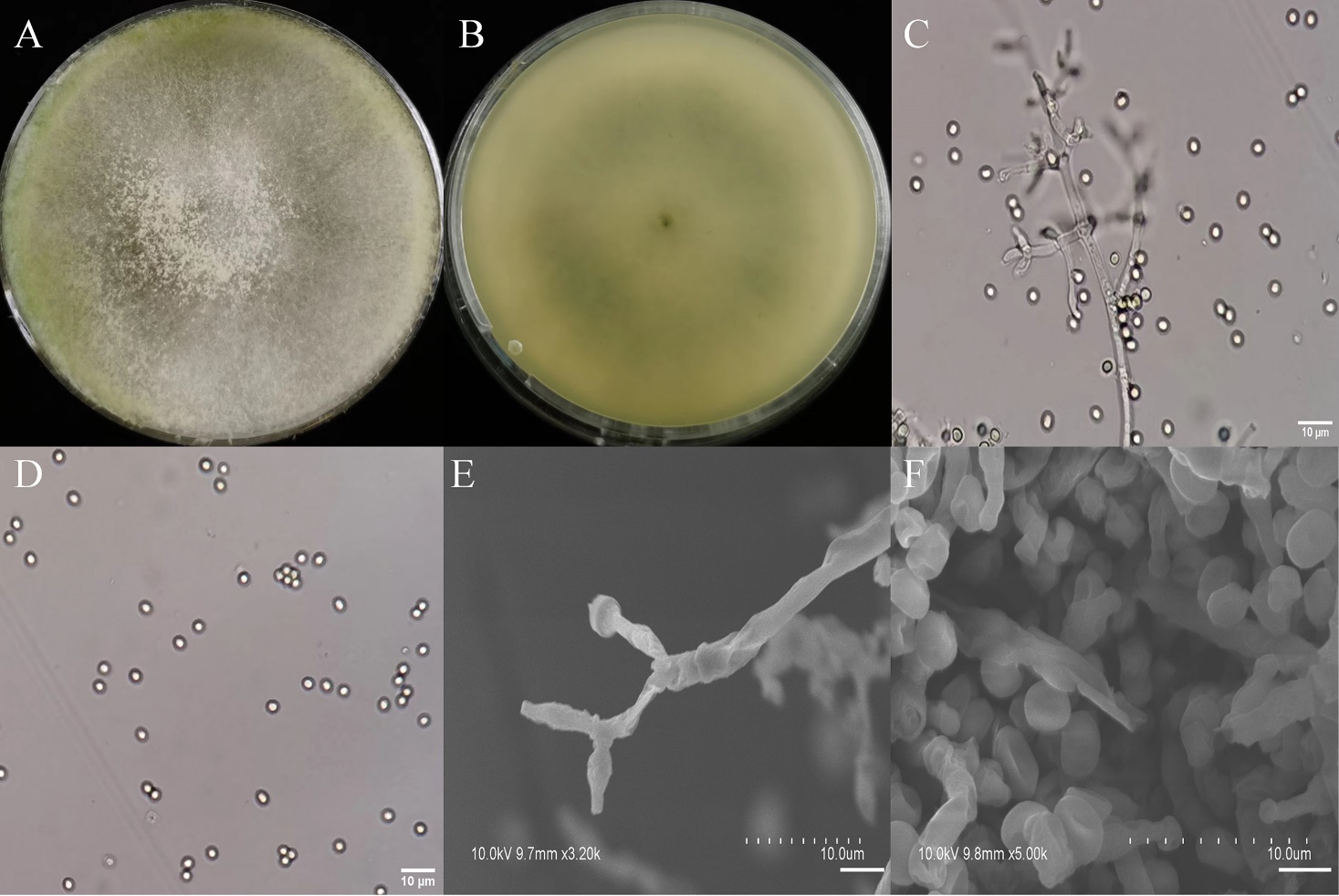
**

**Fig. S8.** Morphology of *T*. *dorotheae* under optical microscope and SEM. (A) Colonial morphology cultured on PDA. (B) Reverse of colony on PDA. (C) Hyphae and conidiophores (OM). (D) Conidia (OM). (E) Conidiophores (SEM). (F) Conidia (SEM).

**
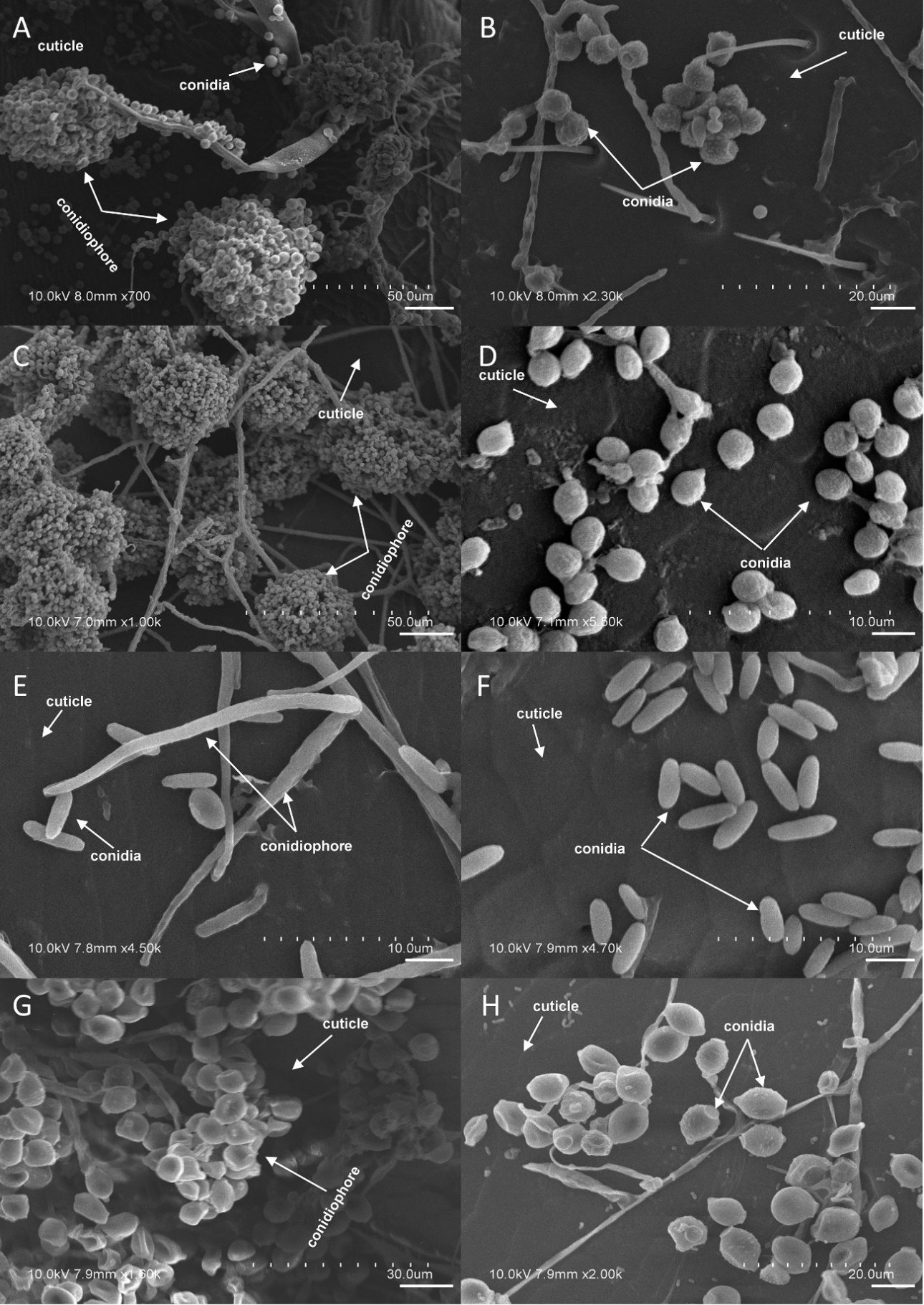
**

**Fig. S9.** *T*. *castaneum* cadavers infected by *A*. *austwickii* (A, B), *B*. *bassiana* (C, D), *L*. *attenuates* (E, F), and *S*. *alboflavescens* (G, H) under SEM. (A, C, E, and G) The conidiophores grown from *T*. *castaneum* cuticle. (B, D, F, and H) Conidia on *T*. *castaneum* cuticle surface.

**
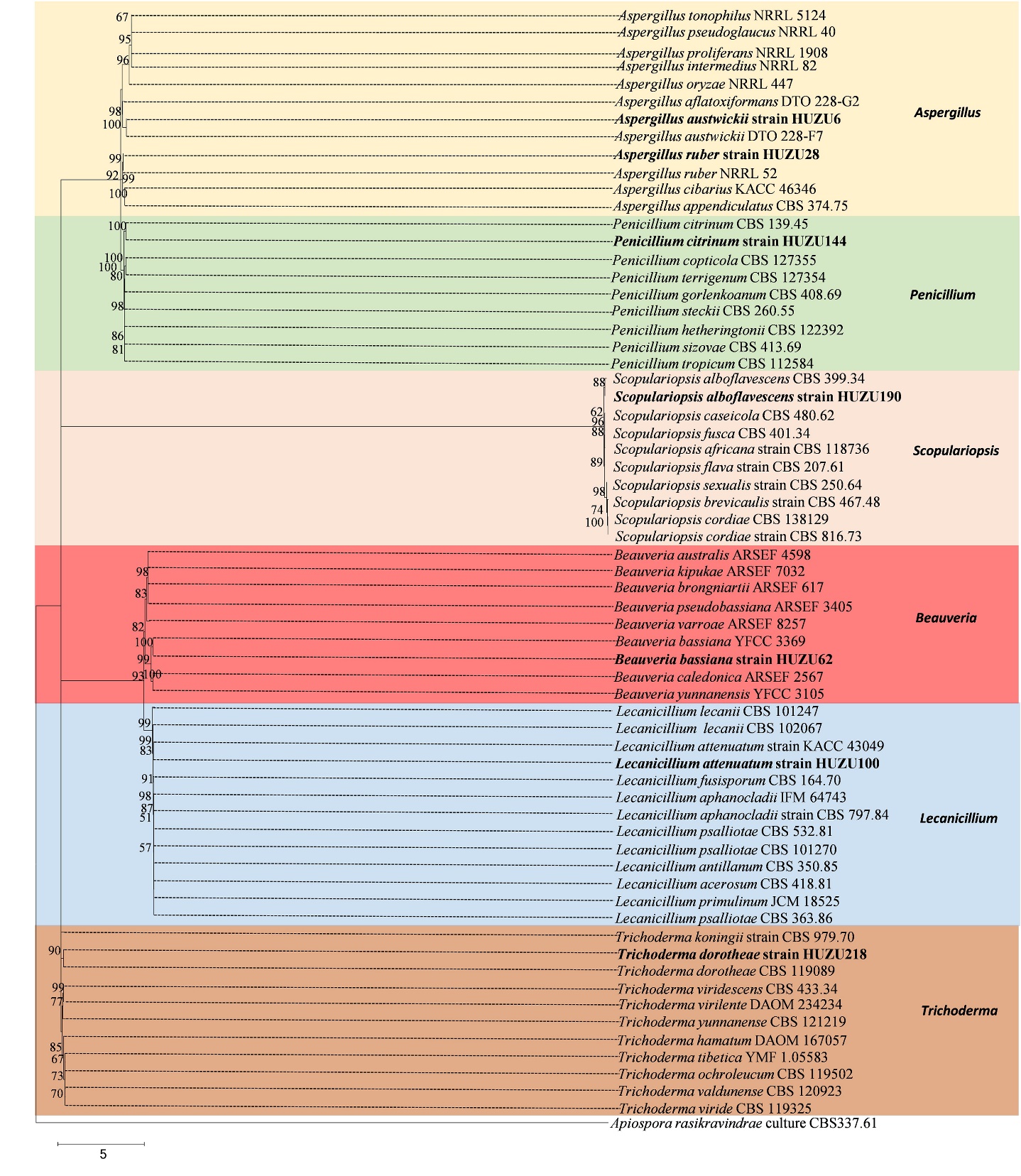
**

**Fig. S10.** Phylogenetic relationships among genera and related species including the seven entomopathogenic fungi inferred from a multigene dataset (ITS, *LSU*, *SSU*, *EF-1α*, *rpb2*, and *β-tubulin*) based on ML analyses. The fungal isolates in this study are in bold. Support values greater than 50% are indicated at the nodes.

## Supplementary Tables

**Table S1** GenBank accession numbers of fungal species sequences used for construction of the phylogenetic tree in Fig. S10.

| Species | Isolate number | GenBank | | | | | |
| --- | --- | --- | --- | --- | --- | --- | --- |
|  |  | ITS | EF | LSU | NS | RPB2 | TUB |
| *Aspergillus austwickii* | DTO 228-F7 | NR_171607 | - | - | MG662391 | MG517893 | MG517702 |
| *Aspergillus aflatoxiformans* | DTO 228-G2 | NR_171606 | - | - | MG662388 | MG517897 | MG517706 |
| *Aspergillus appendiculatus* | CBS 374.75 | NR_135433 | - | - | HE615132 | HE801307 | HE801333 |
| *Aspergillus cibarius* | KACC 46346 | NR_111728 | - | - | JQ918177 | JQ918186 | JQ918180 |
| *Aspergillus intermedius* | NRRL 82 | NR_137448 | - | U29548 | EF652074 | - | EF651892 |
| *Aspergillus oryzae* | NRRL 447 | NR_135395 | - | U28890 | EF661560 | - | EF661483 |
| *Aspergillus pseudoglaucus* | NRRL 40 | NR_135336 | - | U29542 | EF652050 | - | EF651917 |
| *Aspergillus proliferans* | NRRL 1908 | NR_135339 | - | U29564 | EF652064 | - | EF651891 |
| *Aspergillus ruber* | NRRL 52 | NR_131286 | - | U29543 | EF652066 | - | EF651920 |
| *Aspergillus tonophilus* | NRRL 5124 | NR_137450 | - | U29572 | EF652081 | - | EF651919 |
| ***Aspergillus austwickii*** | **HUZU6** | **OP321304** | **OP373162** | **OP352126** | **OP352251** | **OP373163** | **OP373164** |
| ***Aspergillus rube****r* | **HUZU28** | **OP321326** | **OP373165** | **OP352127** | **OP352252** | **OP373166** | **OP373167** |
| *Apiospora rasikravindrae* | CBS337.61 | - | - | KF144961 | KF144914 | - | - |
| *Beauveria australis* | ARSEF 4598 | NR_111597 | HQ880995 | - | HQ880789 | HQ880933 | - |
| *Beauveria bassiana* | YFCC3369 | - | MN576994 | MN576824 | MN576768 | MN576938 | - |
| *Beauveria brongniartii* | ARSEF 617 | NR_111595 | HQ880991 | - | HQ880782 | HQ880926 | - |
| *Beauveria caledonica* | ARSEF 2567 | - | EF469057 | NG_058620 | AF339570 | HQ880961 | EF469134 |
| *Beauveria kipukae* | ARSEF 7032 | NR_111600 | HQ881005 | - | HQ880803 | HQ880947 | - |
| [*Beauveria pseudobassiana*](https://www.ncbi.nlm.nih.gov/nuccore/NR_111598.1) | ARSEF 3405 | NR_111598 | - | - | HQ880792 | HQ880936 | - |
| *Beauveria varroae* | ARSEF 8257 | NR_111599 | HQ881002 | - | HQ880800 | HQ880944 | KJ500383 |
| *Beauveria yunnanensis* | YFCC 3105 | - | MN576999 | MN576829 | MN576773 | MN576943 | - |
| ***Beauveria bassiana*** | **HUZU62** | **OP321360** | **OP373168** | **OP352128** | **OP352253** | **OP373169** | **OP373170** |
| *Lecanicillium acerosum* | CBS 418.81 | - | KM283810 | KM283786 | KM283762 | KM283852 | - |
| *Lecanicillium lecanii* | CBS 101247 | - | DQ522359 | - | - | DQ522466 | DQ522529 |
| *Lecanicillium lecanii* | CBS 102067 | - | KM283818 | KM283795 | KM283771 | KM283860 | - |
| *Lecanicillium antillanum* | CBS 350.85 | NR_111097 | DQ522350 | AF339536 | AF339585 | DQ522450 | DQ522514 |
| *Lecanicillium aphanocladii* | IFM 64743 | - | LC553294 | LC553284 | LC553289 | - | - |
| *Lecanicillium aphanocladii* | CBS 797.84 | - | KM283811 | KM283787 | KM283763 | KM283853 | - |
| *Lecanicillium attenuatum* | KACC 43049 | - | KM283805 | KM283781 | KM283757 | KM283847 | - |
| *Lecanicillium fusisporum* | CBS 164.70 | NR_111100 | KM283817 | KM283793 | KM283769 | KM283858 | - |
| *Lecanicillium psalliotae* | CBS 363.86 | - | EF468784 | AF339559 | AF339608 | - | - |
| *Lecanicillium psalliotae* | CBS 101270 | - | EF469066 | EF469081 | EF469128 | EF469113 | EF469146 |
| *Lecanicillium psalliotae* | CBS 532.81 | - | EF469067 | AF339560 | AF339609 | EF469112 | EF469145 |
| [*Lecanicillium primulinum*](https://www.ncbi.nlm.nih.gov/nuccore/NR_119418.1) | JCM 18525 | NR_119418 | LC557125 | NG_067516 | NG_073501 | - | - |
| ***Lecanicillium attenuatum*** | **HUZU100** | **OP321398** | **OP373171** | **OP352129** | **OP352254** | **OP373159** | **OP373172** |
| *Penicillium citrinum* | CBS 139.45 | NR_121224 | - | MH867647 | MH856132 | JF417416 | GU944545 |
| *Penicillium copticola* | CBS 127355 | NR_121516 | - | - | JN617685 | JN606599 | JN606817 |
| *Penicillium gorlenkoanum* | CBS 408.69 | NR_111484 | - | NG_064072 | GU944581 | JN606601 | GU944520 |
| *Penicillium hetheringtonii* | CBS 122392 | NR_111482 | - | - | GU944558 | JN606606 | GU944538 |
| *Penicillium sizovae* | CBS 413.69 | NR_111487 | - | NG_064073 | GU944588 | JN606603 | GU944535 |
| *Penicillium steckii* | CBS 260.55 | NR_111488 | - | MH869018 | MH857476 | JN606602 | GU944522 |
| *Penicillium tropicum* | CBS 112584 | NR_111485 | - | - | GU944582 | JN606607 | GU944532 |
| *Penicillium terrigenum* | CBS 127354 | NR_121515 | - | MH875974 | MH864538 | JN606600 | JN606810 |
| ***Penicillium citrinum*** | **HUZU144** | **OP321442** | **OP373173** | **OP352130** | **OP352255** | **OP373174** | **OP373160** |
| *Scopulariopsis alboflavescens* | CBS 399.34 | NR_156620 | KX924179 | NG_067347 | KX923956 | - | - |
| *Scopulariopsis africana* | CBS 118736 | KX923954 | KX924176 | NG_058269 | - | - | KX924388 |
| *Scopulariopsis brevicaulis* | CBS 467.48 | - | KX924201 | - | KX923975 | - | KX924408 |
| *Scopulariopsis cordiae* | CBS 138129 | NR_132958 | KX924249 | - | KX924022 | - | KX924456 |
| *Scopulariopsis caseicola* | CBS 480.62 | NR_155811 | KX924247 | NG_069392 | - | - | KX924454 |
| [*Scopulariopsis cordiae*](https://www.ncbi.nlm.nih.gov/nuccore/KX924455.1) | CBS 816.73 | KX924021 | KX924248 | - | - | - | KX924455 |
| *Scopulariopsis fusca* | CBS 401.34 | NR_145258 | JQ434627 | JQ434675 | - | - | JQ434564 |
| *Scopulariopsis flava strain* | CBS 207.61 | - | JQ434598 | JQ434645 | KX924023 | - | JQ434534 |
| *Scopulariopsis sexualis* | CBS 250.64 | KX924024 | KX924251 | - | - | - | KX924458 |
| ***Scopulariopsis alboflavescens*** | **HUZU190** | **OP321488** | **OP373175** | **OP352131** | **OP352256** | **OP373176** | **OP373161** |
| *Trichoderma dorotheae* | CBS 119089 | NR_166014 | - | MH874604 | MH863050 | - | - |
| *Trichoderma hamatum* | DAOM 167057 | NR_134371 | AF534620 | NG_069076 | EU280124 | AF545548 | - |
| *Trichoderma koningii* | CBS 979.70 | - | AY665703 | AF399239 | AF399177 | EU248601 | - |
| *Trichoderma ochroleucum* | CBS 119502 | NR_134401 | FJ860659 | - | - | FJ860556 | - |
| *Trichoderma tibetica* | YMF 1.05583 | NR_176705 | MK779179 | - | MK779177 | MK779178 | - |
| *Trichoderma viridescens* | CBS 433.34 | NR_138429 | AY376048 | NG_069630 | AF456922 | - | - |
| *Trichoderma virilente* | DAOM 234234 | NR_138447 | EU280009 | - | EU280119 | - | - |
| *Trichoderma viride* | CBS 119325 | NR_138441 | DQ672615 | - | - | EU711362 | - |
| *Trichoderma yunnanense* | CBS 121219 | NR_134419 | GU198243 | - | GU198302 | GU198274 | - |
| *Trichoderma valdunense* | CBS 120923 | NR_134418 | FJ860717.1 | - | - | FJ860605 | - |
| ***Trichoderma dorotheae*** | **HUZU218** | **OP321516** | **OP373177** | **OP352132** | **OP352257** | **OP373178** | **OP373179** |

Note: The entomopathogenic fungal species isolated from *M*. *alternatus* in this study are in bold.

**Table S2** Relative abundances (%) of fungal species in each geographical population of *M*. *alternatus*.

| Fungi species | Zhejiang Province | | Sichuan Province | | Fujian Province | | Guangdong Province | | Guangxi Province | |
| --- | --- | --- | --- | --- | --- | --- | --- | --- | --- | --- |
|  | N | % | N | % | N | % | N | % | N | % |
| *Arthrinium rasikravindrae* | 0 | 0 | 1 | 2.78 | 0 | 0 | 0 | 0 | 0 | 0 |
| *Aspergillus austwickii* | 4 | 2.03 | 15 | 41.67 | 3 | 1.74 | 0 | 0 | 3 | 1.86 |
| *Aspergillus ruber* | 0 | 0 | 0 | 0 | 2 | 1.16 | 0 | 0 | 137 | 85.09 |
| *Aspergillus sydowii* | 0 | 0 | 0 | 0 | 50 | 29.07 | 13 | 17.57 | 0 | 0 |
| *Beauveria bassiana* | 2 | 1.02 | 0 | 0 | 0 | 0 | 0 | 0 | 0 | 0 |
| *Cladosporium delicatulum* | 0 | 0 | 0 | 0 | 0 | 0 | 1 | 1.35 | 0 | 0 |
| *Clonostachys aranearum* | 8 | 4.06 | 0 | 0 | 0 |  | 2 | 2.70 | 0 | 0 |
| *Clonostachys eriocamporesiana* | 0 | 0 | 0 | 0 | 0 | 0 | 4 | 5.41 | 0 | 0 |
| *Clonostachys rosea* | 3 | 1.52 | 0 | 0 | 0 | 0 | 0 | 0 | 0 | 0 |
| *Fusarium annulatum* | 19 | 9.64 | 0 | 0 | 6 | 3.49 | 8 | 10.81 | 0 | 0 |
| *Fusarium circinatum* | 2 | 1.02 | 0 | 0 | 0 | 0 | 0 | 0 | 0 | 0 |
| *Fusarium foetens* | 1 | 0.51 | 0 | 0 | 0 | 0 | 0 | 0 | 11 | 6.83 |
| *Fusarium polyphialidicum* | 1 | 0.51 | 0 | 0 | 1 | 0.58 | 0 | 0 | 0 | 0 |
| *Lecanicillium aphanocladii* | 0 | 0 | 0 | 0 | 0 | 0 | 0 | 0 | 2 | 1.24 |
| *Lecanicillium attenuatus* | 50 | 25.38 | 0 | 0 | 4 | 2.33 | 0 | 0 | 0 | 0 |
| *Nigrospora camelliae-sinensis* | 0 | 0 | 1 | 2.78 | 0 | 0. | 0 | 0 | 0 | 0 |
| *Nigrospora musae* | 0 | 0 | 1 | 2.78 | 0 |  | 0 | 0 | 0 | 0 |
| *Penicillium citrinum* | 29 | 14.72 | 0 | 0 | 17 | 9.88 | 17 | 22.97 | 0 | 0 |
| *Penicillium chrysogenum* | 7 | 3.55 | 0 | 0 | 0 | 0 | 0 | 0 | 0 | 0 |
| *Penicillium cairnsense* | 17 | 8.63 | 0 | 0 | 15 | 8.72 | 0 | 0 | 0 | 0 |
| *Penicillium crustosum* | 1 | 0.51 | 0 | 0 | 0 | 0 | 0 | 0 | 0 | 0 |
| *Penicillium meleagrinum var. viridiflavum* | 14 | 7.11 | 0 | 0 | 6 | 3.49 | 2 | 2.70 | 0 | 0 |
| *Penicillium ochrochloron* | 9 | 4.57 | 0 | 0 | 0 | 0 | 1 | 1.35 | 0 | 0 |
| *Penicillium quebecense* | 0 | 0 | 1 | 2.78 | 13 | 7.56 | 13 | 17.57 | 0 | 0 |
| *Pestalotiopsis disseminata* | 0 | 0 | 1 | 2.78 | 0 | 0 | 0 | 0 | 0 | 0 |
| *Pestalotiopsis grevilleae* | 0 | 0 | 1 | 2.78 | 0 | 0 | 0 | 0 | 0 | 0 |
| *Pestalotiopsis microspora* | 6 | 3.05 | 0 | 0 | 0 | 0 | 0 | 0 | 0 | 0 |
| *Purpureocillium*  *lilacinum* | 2 | 1.02 | 0 | 0 | 0 | 0 | 3 | 4.05 | 0 | 0 |
| *Scopulariopsis alboflavescens* | 0 | 0 | 0 | 0 | 54 | 31.40 | 3 | 4.05 | 5 | 3.11 |
| *Syncephalastrummonosporum var. pluriproliferum* | 1 | 0.51 | 0 | 0 | 0 | 0 | 0 | 0 | 0 | 0 |
| *Talaromyces coalescens* | 1 | 0.51 | 0 | 0 | 0 | 0 | 0 | 0 | 0 | 0 |
| *Talaromyces wortmannii* | 0 | 0 | 0 | 0 | 0 | 0 | 2 | 2.70 | 3 | 1.86 |
| *Trichoderma appalachiense* | 1 | 0.51 | 0 | 0 | 0 | 0 | 0 | 0.00 | 0 | 0 |
| *Trichoderma atroviride* | 0 | 0 | 1 | 2.78 | 0 | 0 | 2 | 2.70 | 0 | 0 |
| *Trichoderma dorotheae* | 15 | 7.61 | 7 | 19.44 | 1 | 0.58 | 0 | 0 | 0 | 0 |
| *Trichoderma hispanicum* | 0 | 0 | 1 | 2.78 | 0 | 0 | 0 | 0 | 0 | 0 |
| *Trichoderma lixii* | 0 | 0 | 2 | 5.56 | 0 | 0 | 3 | 4.05 | 0 | 0 |
| *Trichoderma neokoningii* | 4 | 2.03 | 0 | 0 | 0 | 0 | 0 | 0 | 0 | 0 |
| *Trichoderma texanum* | 0 | 0 | 4 | 11.11 | 0 | 0 | 0 | 0 | 0 | 0 |
| Total isolates in each region | 197 | | 36 | | 172 | | 74 | | 161 | |

Note: Relative abundance was calculated as the ratio of the number of isolates of each species (N) to that of total fungal isolates in each geographical region.

**Table S3** Comparison of diversity indices (Mean ± SD) between fungal communities among geographical populations of *M*. *alternatus*.

| Index | Zhejiang Province | Sichuan Province | Fujian Province | Guangdong Province | Guangxi Province | F_4,65_/χ_4_ ^2^ | *P*-value |
| --- | --- | --- | --- | --- | --- | --- | --- |
| Number of species | 3.63±1.71^a^ | 2.43±2.15^a^ | 2.36±1.08^a^ | 2.25±1.75^ab^ | 1.28±0.54^b^ | 23.23 | 0.0001 |
| Shannon diversity (H’) | 0.98±0.54^a^ | 0.58±0.64^a^ | 0.66±0.41^a^ | 0.37±0.55^ab^ | 0.15±0.29^b^ | 24.19 | 0.0001 |
| Simpson’s diversity (1-D) | 0.53±0.27^a^ | 0.33±0.33^ab^ | 0.41±0.24^ab^ | 0.19±0.27^b^ | 0.10±0.19^c^ | 8.89 | 0.0001 |
| Dominance (D) | 0.47±0.27^c^ | 0.67±0.33^ab^ | 0.59±0.24^b^ | 0.81±0.27^ab^ | 0.90±0.19^a^ | 8.89 | 0.0001 |
| Buzas and Gibson's evenness  (e^H’^/S) | 0.86±0.11^b^ | 0.95±0.09^ab^ | 0.92±0.12^ab^ | 0.84±0.20^b^ | 0.97±0.08^a^ | 16 | 0.003 |

Note: In each index, groups sharing the same letter are not significantly different.

**Table S4** Similarity coefficient of fungal community compositions among *M*. *alternatus* populations.

| Sampling region | Zhejiang Province | Sichuan Province | Fujian Province | Guangdong Province | Guangxi Province |
| --- | --- | --- | --- | --- | --- |
| Zhejiang Province | - |  |  |  |  |
| Sichuan Province | 0.12 | - |  |  |  |
| Fujian Province | 0.24 | 0.25 | - |  |  |
| Guangdong Province | 0.33 | 0.23 | 0.46 | - |  |
| Guangxi Province | 0.14 | 0.11 | 0.33 | 0.20 | - |

Note: When 0 ＜ Cs ＜ 0.25, it is very dissimilar; when 0.25 ≤ Cs ＜ 0.50, it is medium dissimilar; when 0.5 ≤ Cs ＜ 0.75, it is medium similar; when 0.75 ≤ Cs ＜ 1.00, it is very similar.

**Table S5** Significance test using one-way PERMANOVA between different *M*. *alternatus* geographical populations by pair-wise comparisons.

| Sampling area | Zhejiang province | Sichuan province | Fujian province | Guangdong province | Guangxi province |
| --- | --- | --- | --- | --- | --- |
| Zhejiang province | - |  |  |  |  |
| Sichuan province | 3.242/0.009 | - |  |  |  |
| Fujian province | 5.507/0.002 | 5.146/0.005 | - |  |  |
| Guangdong province | 2.162/0.001 | 2.206/0.085 | 1.549/1.000 | - |  |
| Guangxi province | 21.540/0.002 | 16.780/0.001 | 22.980/0.001 | 12.260/0.001 | - |

Note: *F* values/*P* values.

**Table S6** Relative abundances (%) of fungal species in each body position of *M*. *alternatus*.

| species | Antenna | | Head | | [Thorax](javascript:;) | | [Abdomen](javascript:;) | | [Wing](javascript:;) | | [Foot](javascript:;) | | Egg | |
| --- | --- | --- | --- | --- | --- | --- | --- | --- | --- | --- | --- | --- | --- | --- |
|  | N | % | N | % | N | % | N | % | N | % | N | % | N | % |
| *Arthrinium rasikravindrae* | 0 | 0 | 0 | 0 | 0 | 0 | 1 | 0.76 | 0 | 0 | 0 | 0 | 0 | 0 |
| *Aspergillus austwickii* | 5 | 4.76 | 1 | 1.75 | 2 | 2.94 | 2 | 1.53 | 5 | 3.60 | 8 | 6.25 | 2 | 16.67 |
| *Aspergillus ruber* | 27 | 25.71 | 8 | 14.04 | 11 | 16.18 | 26 | 19.85 | 30 | 21.58 | 37 | 28.91 | 0 | 0 |
| *Aspergillus sydowii* | 6 | 5.71 | 6 | 10.53 | 7 | 10.29 | 14 | 10.69 | 20 | 14.39 | 10 | 7.81 | 0 | 0 |
| *Beauveria bassiana* | 2 | 1.90 | 0 | 0 | 0 | 0 | 0 | 0 | 0 | 0 | 0 | 0 | 0 | 0 |
| *Cladosporium delicatulum* | 0 | 0 | 0 | 0 | 0 | 0 | 0 | 0 | 0 | 0 | 1 | 0.78 | 0 | 0 |
| *Clonostachys aranearum* | 4 | 3.81 | 0 | 0 | 1 | 1.47 | 0 | 0 | 2 | 1.44 | 3 | 2.34 | 0 | 0 |
| *Clonostachys eriocamporesiana* | 2 | 1.90 | 0 | 0 | 0 | 0 | 0 | 0 | 1 | 0.72 | 1 | 0.78 | 0 | 0 |
| *Clonostachys rosea* | 1 | 0.95 | 0 | 0 | 0 | 0 | 2 | 1.53 | 0 | 0 | 0 | 0 | 0 | 0 |
| *Fusarium annulatum* | 7 | 6.67 | 4 | 7.02 | 4 | 5.88 | 5 | 3.82 | 2 | 1.44 | 7 | 5.47 | 4 | 33.33 |
| *Fusarium circinatum* | 0 | 0 | 0 | 0 | 0 | 0 | 1 | 0.76 | 1 | 0.72 | 0 | 0 | 0 | 0 |
| *Fusarium foetens* | 1 | 0.95 | 1 | 1.75 | 2 | 2.94 | 2 | 1.53 | 3 | 2.16 | 3 | 2.34 | 0 | 0 |
| *Fusarium polyphialidicum* | 0 | 0 | 0 | 0 | 1 | 1.47 | 0 | 0 | 1 | 0.72 | 0 | 0 | 0 | 0 |
| *Lecanicillium aphanocladii* | 0 | 0 | 0 | 0 | 0 | 0 | 0 | 0 | 1 | 0.72 | 1 | 0.78 | 0 | 0 |
| *Lecanicillium attenuatus* | 10 | 9.52 | 7 | 12.28 | 5 | 7.35 | 16 | 12.21 | 8 | 5.76 | 7 | 5.47 | 1 | 8.33 |
| *Nigrospora camelliae-sinensis* | 0 | 0 | 0 | 0 | 1 | 1.47 | 0 | 0 | 0 | 0 | 0 | 0 | 0 | 0 |
| *Nigrospora musae* | 0 | 0 | 0 | 0 | 0 | 0 | 0 | 0 | 1 | 0.72 | 0 | 0 | 0 | 0 |
| *Penicillium citrinum* | 12 | 11.43 | 7 | 12.28 | 3 | 4.41 | 15 | 11.45 | 14 | 10.07 | 12 | 9.38 | 0 | 0 |
| *Penicillium chrysogenum* | 1 | 0.95 | 1 | 1.75 | 0 | 0 | 3 | 2.29 | 1 | 0.72 | 1 | 0.78 | 0 | 0 |
| *Penicillium cairnsense* | 8 | 7.62 | 3 | 5.26 | 5 | 7.35 | 4 | 3.05 | 4 | 2.88 | 6 | 4.69 | 2 | 16.67 |
| *Penicillium crustosum* | 0 | 0 | 0 | 0 | 0 | 0 | 1 | 0.76 | 0 | 0 | 0 | 0 | 0 | 0 |
| *Penicillium meleagrinum var. viridiflavum* | 3 | 2.86 | 4 | 7.02 | 5 | 7.35 | 1 | 0.76 | 5 | 3.60 | 4 | 3.13 | 0 | 0 |
| *Penicillium ochrochloron* | 1 | 0.95 | 1 | 1.75 | 1 | 1.47 | 2 | 1.53 | 3 | 2.16 | 0 | 0 | 2 | 16.67 |
| *Penicillium quebecense* | 2 | 1.90 | 4 | 7.02 | 3 | 4.41 | 6 | 4.58 | 6 | 4.32 | 5 | 3.91 | 1 | 8.33 |
| *Pestalotiopsis disseminata* | 0 | 0 | 0 | 0 | 0 | 0 | 1 | 0.76 | 0 | 0 | 0 | 0 | 0 | 0 |
| *Pestalotiopsis grevilleae* | 0 | 0 | 0 | 0 | 0 | 0 | 1 | 0.76 | 0 | 0 | 0 | 0 | 0 | 0 |
| *Pestalotiopsis microspora* | 1 | 0.95 | 1 | 1.75 | 0 | 0 | 2 | 1.53 | 1 | 0.72 | 1 | 0.78 | 0 | 0 |
| *Purpureocillium*  *lilacinum* | 0 | 0 | 0 | 0 | 2 | 2.94 | 0 | 0 | 0 | 0 | 3 | 2.34 | 0 | 0 |
| *Scopulariopsis alboflavescens* | 5 | 4.76 | 8 | 14.04 | 6 | 8.82 | 17 | 12.98 | 16 | 11.51 | 10 | 7.81 | 0 | 0 |
| *Syncephalastrummonosporum var. pluriproliferum* | 0 | 0 | 0 | 0 | 0 | 0 | 0 | 0 | 0 | 0 | 1 | 0.78 | 0 | 0 |
| *Talaromyces coalescens* | 0 | 0 | 0 | 0 | 0 | 0 | 0 | 0 | 1 | 0.72 | 0 | 0 | 0 | 0 |
| *Talaromyces wortmannii* | 0 | 0 | 0 | 0 | 2 | 2.94 | 0 | 0 | 3 | 2.16 | 0 | 0 | 0 | 0 |
| *Trichoderma appalachiense* | 0 | 0 | 0 | 0 | 1 | 1.47 | 0 | 0 | 0 | 0 | 0 | 0 | 0 | 0 |
| *Trichoderma atroviride* | 0 | 0 | 0 | 0 | 1 | 1.47 | 1 | 0.76 | 1 | 0.72 | 0 | 0 | 0 | 0 |
| *Trichoderma dorotheae* | 4 | 3.81 | 1 | 1.75 | 3 | 4.41 | 5 | 3.82 | 4 | 2.88 | 6 | 4.69 | 0 | 0 |
| *Trichoderma hispanicum* | 1 | 0.95 | 0 | 0 | 0 | 0 | 0 | 0 | 0 | 0 | 0 | 0 | 0 | 0 |
| *Trichoderma lixii* | 1 | 0.95 | 0 | 0 | 0 | 0 | 1 | 0.76 | 3 | 2.16 | 0 | 0 | 0 | 0 |
| *Trichoderma neokoningii* | 1 | 0.95 | 0 | 0 | 1 | 1.47 | 0 | 0 | 1 | 0.72 | 1 | 0.78 | 0 | 0 |
| *Trichoderma texanum* | 0 | 0 | 0 | 0 | 1 | 1.47 | 2 | 1.53 | 1 | 0.72 | 0 | 0 | 0 | 0 |
| Total isolates in each body position | 105 | | 57 | | 68 | | 131 | | 139 | | 128 | | 12 | |

Note: Relative abundance was calculated as the ratio of the number of isolates of each species (N) to that of total fungal isolates in each body position.

**Table S7** Relative abundances (%) of fungal species in each gender of *M*. *alternatus*.

| Fungi specie | Male | | Female | |
| --- | --- | --- | --- | --- |
|  | N | % | N | % |
| *Arthrinium rasikravindrae* | 0 | 0 | 1 | 0.22 |
| *Aspergillus austwickii* | 5 | 2.67 | 20 | 4.42 |
| *Aspergillus ruber* | 25 | 13.37 | 114 | 25.17 |
| *Aspergillus sydowii* | 25 | 13.37 | 38 | 8.39 |
| *Beauveria bassiana* | 0 | 0 | 2 | 0.44 |
| *Cladosporium delicatulum* | 1 | 0.53 | 0 | 0 |
| *Clonostachys aranearum* | 8 | 4.28 | 2 | 0.44 |
| *Clonostachys eriocamporesiana* | 4 | 2.14 | 0 | 0 |
| *Clonostachys rosea* | 0 | 0 | 3 | 0.66 |
| *Fusarium annulatum* | 15 | 8.02 | 18 | 3.97 |
| *Fusarium circinatum* | 0 | 0 | 2 | 0.44 |
| *Fusarium foetens* | 0 | 0 | 12 | 2.65 |
| *Fusarium polyphialidicum* | 0 | 0 | 2 | 0.44 |
| *Lecanicillium aphanocladii* | 2 | 1.07 | 0 | 0 |
| *Lecanicillium attenuatus* | 10 | 5.35 | 44 | 9.71 |
| *Nigrospora camelliae-sinensis* | 0 | 0 | 1 | 0.22 |
| *Nigrospora musae* | 0 | 0 | 1 | 0.22 |
| *Penicillium citrinum* | 33 | 17.65 | 30 | 6.62 |
| *Penicillium chrysogenum* | 0 | 0 | 7 | 1.55 |
| *Penicillium cairnsense* | 7 | 3.74 | 25 | 5.52 |
| *Penicillium crustosum* | 1 | 0.53 | 0 | 0 |
| *Penicillium meleagrinum var. viridiflavum* | 4 | 2.14 | 18 | 3.97 |
| *Penicillium ochrochloron* | 5 | 2.67 | 5 | 1.10 |
| *Penicillium quebecense* | 0 | 0 | 27 | 5.96 |
| *Pestalotiopsis disseminata* | 0 | 0 | 1 | 0.22 |
| *Pestalotiopsis grevilleae* | 0 | 0 | 1 | 0.22 |
| *Pestalotiopsis microspora* | 0 | 0 | 6 | 1.32 |
| *Purpureocillium lilacinum* | 3 | 1.60 | 2 | 0.44 |
| *Scopulariopsis alboflavescens* | 18 | 9.63 | 44 | 9.71 |
| *Syncephalastrum monosporum var. pluriproliferum* | 1 | 0.53 | 0 | 0 |
| *Talaromyces coalescens* | 0 | 0 | 1 | 0.22 |
| *Talaromyces wortmannii* | 5 | 2.67 | 0 | 0 |
| *Trichoderma appalachiense* | 0 | 0 | 1 | 0.22 |
| *Trichoderma atrovirid* | 2 | 1.07 | 1 | 0.22 |
| *Trichoderma dorotheae* | 7 | 3.74 | 16 | 3.53 |
| *Trichoderma hispanicum* | 0 | 0 | 1 | 0.22 |
| *Trichoderma lixii* | 2 | 1.07 | 3 | 0.66 |
| *Trichoderma neokoningii* | 4 | 2.14 | 0 | 0 |
| *Trichoderma texanum* | 0 | 0 | 4 | 0.88 |
| Total isolates in each gender | 187 | | 453 | |

Note: Relative abundance was calculated as the ratio of the number of isolates of each species (N) to that of total fungal isolates in each gender.
